# FADS2‐STING Signaling in Sertoli Cells Orchestrates Collagen Deposition and Germ Cell Necroptosis in Idiopathic Non‐Obstructive Azoospermia

**DOI:** 10.1002/advs.78583

**Published:** 2026-11-09

**Authors:** Jiajing Lin, Hongfei Ke, Xiaomin Li, Man Wu, Zhengyan Wu, Zhiying Huang, Feixue Han, Dejian Pang, Shuyan Chen, Tianwen Peng, Yu Lan, Xin Fu, Yingying Zhang, Hyeon Jeong Lee, Zhicong Chen, Geng An

**Affiliations:** ^1^ National Health Commission (NHC) KeyLaboratory of Male Reproduction and Genetics Guangdong Provincial Reproductive Science Institute (Guangdong Provincial Fertility Hospital) Guangzhou China; ^2^ Department of Obstetrics and Gynecology Center for Reproductive Medicine Guangdong Provincial Key Laboratory of MajorObstetric Diseases Guangdong Provincial Clinical Research Center For Obstetrics and Gynecology Guangdong Hong Kong‐Macao Greater Bay Area Higher Education Joint Laboratory of Maternal‐Fetal Medicine The Third Affiliated Hospital|Guangzhou Medical University Guangzhou China; ^3^ College of Biomedical Engineering & Instrument Science Key Laboratory for Biomedical Engineering of Ministry of Education Zhejiang University Hangzhou China; ^4^ The First People's Hospital of Zhaoqing Zhaoqing China

**Keywords:** FADS2, idiopathic non‐obstructive azoospermia, necroptosis, peritubular fibrosis, sertoli cells, STING

## Abstract

Idiopathic non‐obstructive azoospermia (iNOA) is a severe form of male infertility with poorly defined etiology. While dysregulated lipid metabolism is a hallmark of spermatogenic failure, the molecular drivers linking lipid remodeling to testicular niche deterioration remain elusive. Here, integrated testicular lipidomics and single‐cell transcriptomics reveal a metabolic shift from polyunsaturated fatty acid (PUFA)‐enriched toward more saturated phospholipids in iNOA testes, accompanied by a selective downregulation of fatty acid desaturase 2 (FADS2) in Sertoli cells. Mechanistically, we demonstrate that FADS2 deficiency acts as a metabolic trigger for stimulator of interferon genes (STING) signaling, which orchestrates a transcriptional reprogramming in Sertoli cells. This process promotes aberrant collagen metabolic reprogramming and peritubular fibrosis while concurrently sensitizing germ cells to interferon‐mediated necroptosis. Importantly, restoring FADS2‐dependent metabolites (γ‐linolenic acid and docosahexaenoic acid) attenuates STING activation and collagen deposition and partially restores spermatogenesis in vivo. Collectively, these findings define the pathological relevance of Sertoli cell‐associated FADS2‐PUFA remodeling and STING‐associated inflammatory activation in iNOA, supporting PUFA replenishment as a potential strategy to mitigate structural niche pathology and germ‐cell vulnerability.

## Graphical abstract

In Sertoli cells of iNOA testes, downregulation of FADS2 activates STING, promoting collagen deposition and peritubular fibrosis while increasing germ‐cell susceptibility to necroptosis. Supplementation with FADS2‐derived PUFAs (GLA and DHA) inhibits STING, alleviates collagen accumulation, and facilitates the recovery of spermatogenesis. (Figure was created with the help of BioRender.com).

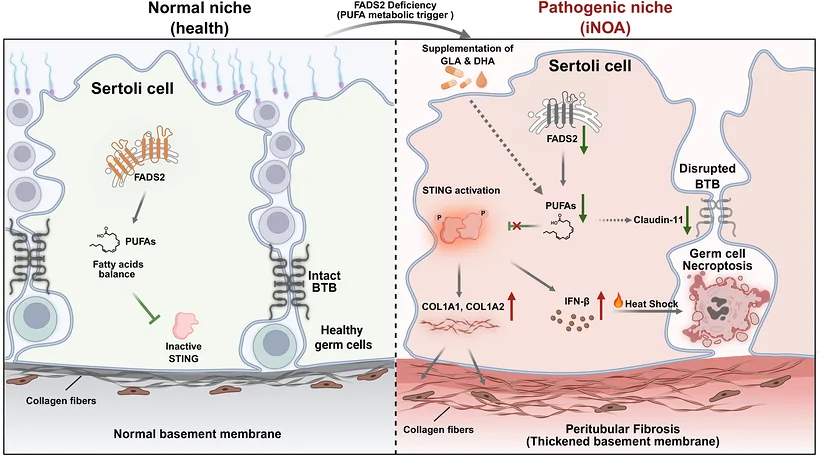

## 1 Introduction

Non‐obstructive azoospermia (NOA) is one of the most severe forms of male infertility, accounting for about 10%–15% of infertile men [1]. Approximately 70% of NOA cases lack an identifiable etiology and are therefore classified as idiopathic NOA (iNOA) [2]. Owing to the unresolved causes and pathogenic mechanisms, effective disease‐modifying therapies for iNOA remain unavailable, and sperm retrieval largely relies on microdissection testicular sperm extraction (micro‐TESE) [3]. In parallel, increasing evidence from multi‐omics studies has begun to implicate lipid metabolic dysregulation in iNOA [4, 5], including marked alterations in energy and lipid metabolism across key somatic compartments such as Leydig cells and Sertoli cells [6, 7]. Nonetheless, how these metabolic abnormalities translate into germ‐cell depletion and sustained spermatogenic failure in iNOA remains largely unknown, highlighting an urgent need to define actionable metabolic determinants within the testicular microenvironment.

Lipid metabolism, particularly the metabolism of polyunsaturated fatty acids (PUFAs), has emerged as a critical determinant of spermatogenesis, a process that depends on dynamic lipid remodeling and an adequate supply of unsaturated fatty acids [8]. PUFAs contain multiple carbon–carbon double bonds and are integral to the lipid composition of cellular membranes, maintaining membrane stability while also acting as signaling molecules that regulate diverse cellular processes [9, 10]. Based on the position of the first double bond from the methyl end, PUFAs are generally categorized into n‐3 and n‐6 families [11]. Notably, the male reproductive system exhibits a distinctive fatty‐acid composition for sperm production [12]. Accordingly, testicular lipid imbalance disrupts membrane homeostasis, energy metabolism, and signal transduction, thereby promoting apoptosis, impairing spermatogenesis, and leading to abnormal sperm morphology and function [13]. Consistent with this, supplementation with unsaturated fatty acids has been reported to improve semen parameters [14]. While prior lipidomic and clinical observations in severe spermatogenic failure have suggested a reduction in PUFA‐enriched phospholipids, these descriptive findings provide limited mechanistic insight [15, 16]. Crucially, the enzymatic determinants of PUFA remodeling and the downstream effector programs that connect altered PUFA desaturation to a deteriorating spermatogenic niche in iNOA remain poorly understood.

Fatty acid desaturase 2 (FADS2) is a key enzyme in the biosynthetic pathway of both n‐3 and n‐6 PUFAs [17]. FADS2 catalyzes the initial desaturation of essential fatty acids and is widely regarded as the rate‐limiting step in PUFA biosynthesis [18, 19]. Working in concert with elongation of very long‐chain fatty acid proteins (ELOVLs) and fatty acid desaturase 1 (FADS1), FADS2 contributes to the generation of long‐chain PUFAs such as arachidonic acid (AA) and docosahexaenoic acid (DHA) [20, 21]. Notably, FADS2 has also been suggested to possess Δ4‐desaturase activity that facilitates DHA synthesis, thereby expanding its functional scope [22]. In the male reproductive system, FADS2‐mediated PUFA supply is essential for maintaining testicular homeostasis. Loss of *Fads2* in mice disrupts the blood‐testis barrier and causes severe spermatogenic failure, ultimately leading to male infertility [23]. Exogenous supplementation with AA and DHA can remodel membrane lipid composition and promote recovery of barrier integrity and spermatogenic function [24]. Although the essential role of the FADS2‐PUFA axis in spermatogenesis has been well established in genetic models, the expression and functional status of FADS2 in human iNOA testes, the associated PUFA remodeling patterns, and their pathological significance remain systematically undefined.

Here, we profiled the PUFA‐associated lipid landscape in testes from patients with iNOA and investigated how FADS2‐associated metabolic remodeling is linked to Sertoli cell dysfunction and spermatogenic niche deterioration. We found that iNOA testes displayed a shift from unsaturated toward more saturated phospholipids, accompanied by a marked downregulation of FADS2 in Sertoli cells. Mechanistically, employing both cell‐based and ex vivo human testicular tissue culture models, we further linked impaired FADS2 function to STING‐TBK1 activation. This signaling axis was associated with transcriptional reprogramming in Sertoli cells, peritubular collagen remodeling, and increased germ‐cell susceptibility to stress‐induced necroptotic signaling. Furthermore, we showed that supplementation with FADS2‐derived PUFA products, γ‐linolenic acid (GLA) and DHA, attenuated STING‐TBK1 activation and mitigated these pathological alterations. Together, these findings define the pathological relevance of Sertoli cell‐associated FADS2‐PUFA remodeling in iNOA and support PUFA replenishment as a potential strategy to attenuate STING‐associated inflammatory activation and collagen remodeling within the spermatogenic niche.

## 2 Results

### 2.1 Dysregulated Unsaturated Phospholipid Metabolism Coincides With Reduced Sertoli Cell FADS2 Expression in iNOA

Given the central role of unsaturated fatty acids in spermatogenesis [25, 26], we first investigated whether the lipid landscape is perturbed in the testicular tissue of iNOA patients (Figure S1A). In both controls and iNOA testes, phospholipids dominated the lipidome (>85% of total lipids) (Figure 1A). However, among the differentially expressed phospholipids, the majority of downregulated species contained PUFAs, whereas the upregulated species were enriched in phospholipids containing saturated fatty acids or oxidized phospholipids (Figure 1B and Figure S1B). When stratified by saturation index (total number of carbon double bonds across both acyl chains), the proportion of unsaturated phospholipids was significantly reduced in iNOA relative to controls, with a concomitant increase in more saturated species (Figure 1C). At the acyl‐chain level, lipids containing 18:3, 20:3, and 22:6 were notably decreased in iNOA (Figure S1C). Validation at the fatty acid desaturases in human testicular tissue further showed a confirmed consistent, significant downregulation of FADS2 in iNOA, whereas stearoyl‐CoA desaturase (SCD) and FADS1 levels remained largely unchanged (Figure 1D). Immunohistochemistry localized this alteration to the seminiferous tubules, where FADS2 staining intensity was significantly lower in iNOA samples (Figure 1E,F). Consistently, *FADS2* mRNA was also reduced in iNOA testes (Figure 1G).

**FIGURE 1:**
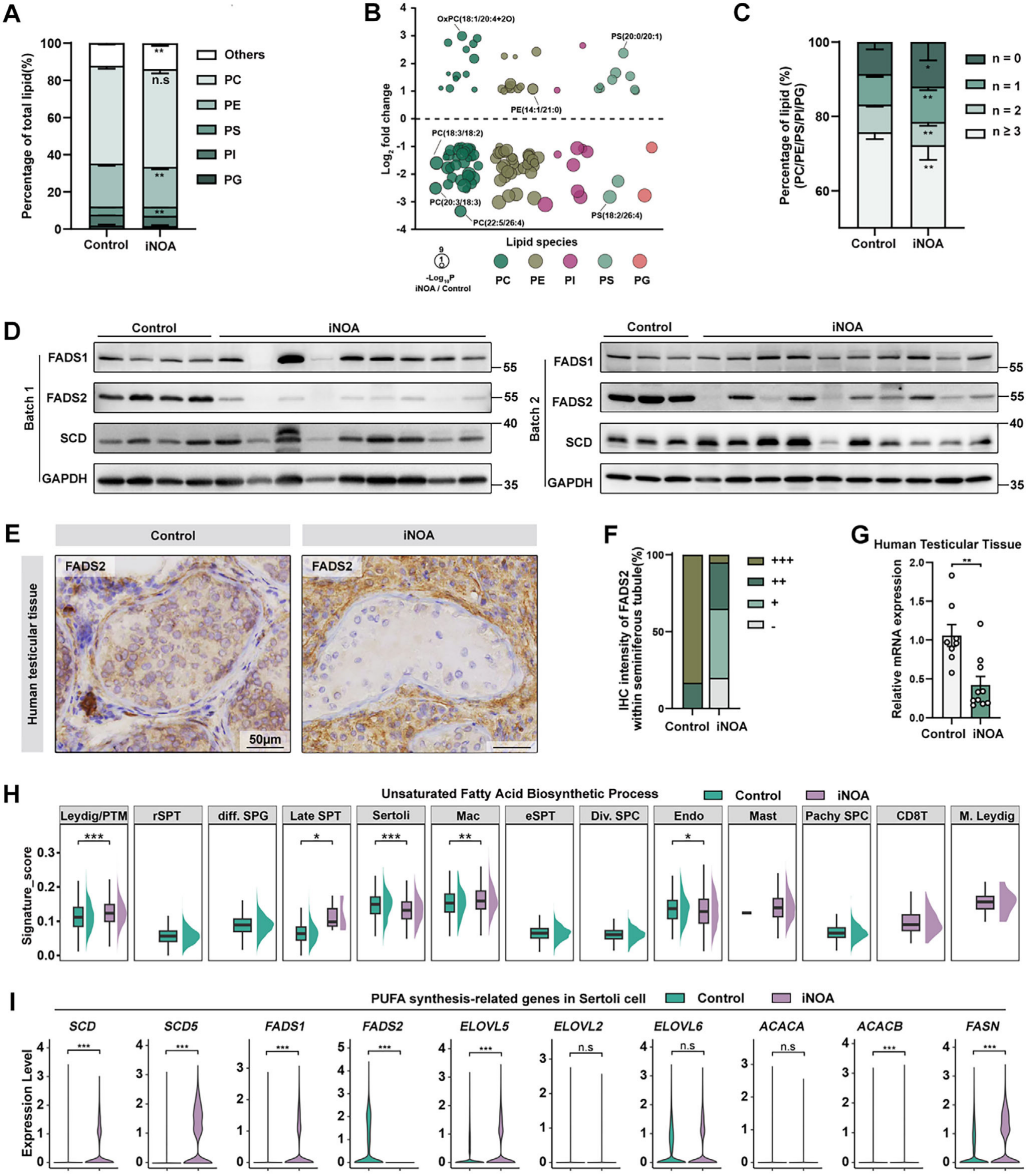
Dysregulated PUFA metabolism and reduced Sertoli cell FADS2 expression in human iNOA testes. (A) Proportions of lipid classes in human testicular tissue from control (*n* = 11) and iNOA group (*n* = 17). Others primarily denote pooled low‐abundance lipid classes, including SM, CL, TAG, CE, DAG, PA, Cer, GlcCer, MGDG, FA, FAHFA, Acar, BMP, HMBP, GM3, PMeOH, PEIOH, AcylglcADG and GlcADG. PC: Phosphatidylcholine; PE: Phosphatidylethanolamine; PS: Phosphatidylserine; PI: Phosphatidylinositol; PG: Phosphatidylglycerol. Data are expressed as mean ± SEM. (B) Lipidomics analysis of phospholipids in human testicular tissue from control and iNOA. Values are shown as log_2_fold change relative to control. Each dot represents one lipid species. Dot color shows lipid class, and dot size indicates significance (‐Lg*P* value). (C) Percentage of phospholipids with different saturation indexes in control and iNOA group. Saturation index (n) of phospholipids was calculated by the total number of carbon double bonds contained in two fatty acyl chains. n = 0 represents saturated phospholipids. Data are expressed as mean ± SEM. (D) Western blot of fatty acid desaturase (FADS1, FADS2 and SCD) of human testicular tissue from control (*n* = 7) and iNOA patients (*n* = 19). (E,F) Representative immunohistochemistry images staining for FADS2 in human testicular tissues (E) and quantification of FADS2 intensity within seminiferous tubules (F) from control (*n* = 6) and iNOA (*n* = 20). Scale bars indicate 50 µm. (G) Relative mRNA expression of *FADS2* in human testicular tissue between control (*n* = 8) and iNOA (*n* = 10). Data are expressed as mean ± SEM. (H) Violin plots showing signature score for the unsaturated fatty acid biosynthetic process in control and iNOA groups, derived from scRNA‐seq datasets GSE149512 and GSE112013. (I) Violin plot showing expression of polyunsaturated fatty acid (PUFA) synthesis‐related genes in Sertoli cells between the control and iNOA groups. *P* values in (A, C, and G) were determined by unpaired Student's *t*‐test. ^*^ *p* < 0.05, ^**^ *p* < 0.01, ^***^ *p* < 0.001, n.s, not significant.

To delineate the cellular contribution to this metabolic deficit, we integrated two scRNA‐seq datasets (GSE149512 and GSE112013) for downstream analyses (Figure S2A,B). We calculated signature scores for the unsaturated fatty‐acid biosynthetic process across cell types identified by established markers (Figure S2C). Leydig cells, late spermatids, endothelial cells, and Sertoli cells exhibited relatively higher unsaturated fatty acid biosynthesis signature scores, indicating a testicular compartment enriched for this pathway. Importantly, the signature score in Sertoli cells was significantly diminished in iNOA, suggesting compromised unsaturated fatty‐acid synthesis capacity specifically in this compartment (Figure 1H). Focusing on Sertoli cells, *FADS2* was markedly decreased, *SCD* showed a mild decrease, and *FADS1* increased at the transcript level in iNOA (Figure 1I). Together, these data reveal a deficit in unsaturated phospholipids in iNOA and implicate reduced *FADS2* expression in Sertoli cells as a potential contributor to this metabolic alteration.

### 2.2 FADS2 Inhibition Impairs the Cellular Functions of Sertoli Cells

To investigate the functional consequences of FADS2 loss, we assessed the viability of TM4 cells following siRNA‐mediated *Fads2* knockdown under distinct serum conditions (Figure S4A,B). Under low‐serum conditions (0.5% FBS), *Fads2* knockdown significantly reduced TM4 cell viability at 60 h compared with controls (Figure 2A). Consistently, pharmacological inhibition of FADS2 using SC26196 dose‐ and time‐dependently decreased TM4 cell viability (Figure 2B). To further validate this effect in a human primary cell, primary human Sertoli cells (hSCs) were isolated (Figure S3A) and treated with SC26196, which similarly reduced cell viability at 48 h (Figure 2C). In parallel, both knockdown and chemical inhibition of FADS2 increased the ROS level in TM4 cells, indicative of oxidative stress (Figure 2D and Figure S4C,D).

**FIGURE 2:**
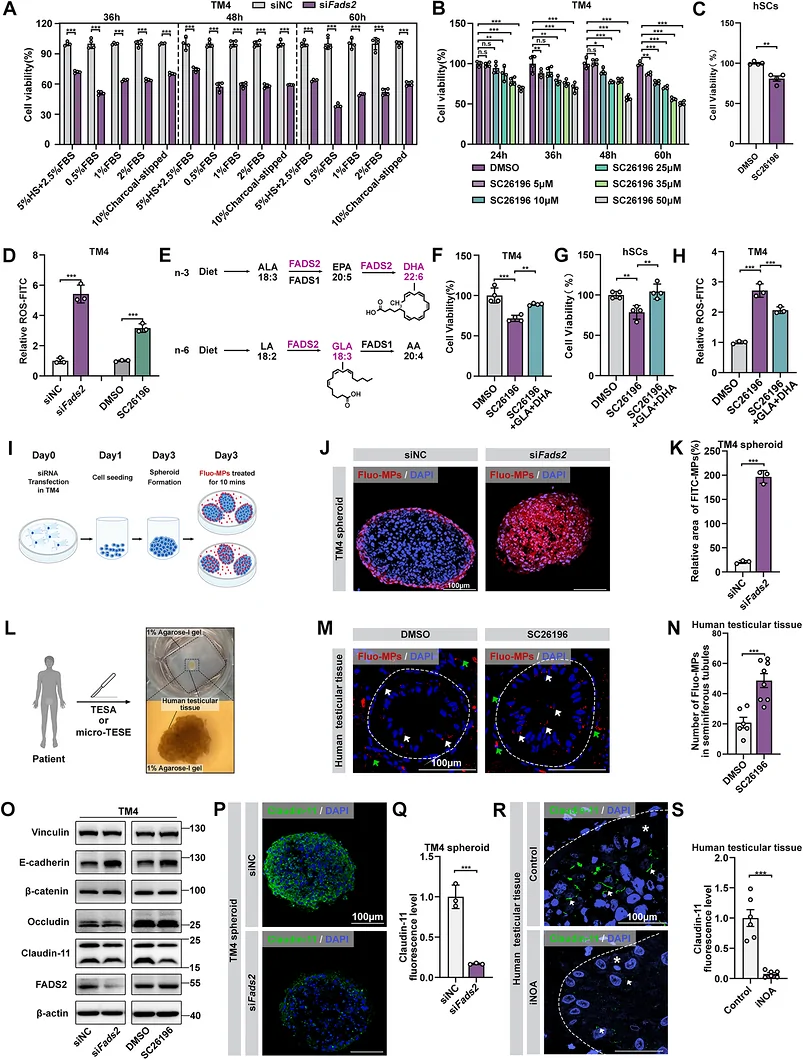
FADS2 deficiency impairs Sertoli cell viability and blood‐testis barrier integrity. (A) Cell viability at 36, 48, and 60 h was measured by Cell Counting Kit‐8 (CCK‐8) in *Fads2*‐knockdown TM4 under culture conditions with varying serum percentages. (B) Cell viability at 24, 36, 48, and 60 h in TM4 cells treated with 5, 10, 25, 35, 50 µM SC26196 (FADS2 inhibitor) in culture medium containing 0.5% FBS. (C) Cell viability at 48 h in human Sertoli cells (hSCs) treated with 10 µM SC26196. (D) Proportion of ROS‐positive TM4 cells after 60 h of *Fads2*‐knockdown or FADS2 inhibition, measured by flow cytometry. (E) Simplified schematic of omega‐3 (n‐3) and omega‐6 (n‐6) fatty acid desaturation pathway. Steps labeled FADS2 or FADS1 denote reactions requiring FADS2‐dependent delta‐6 desaturase (Δ6D) or FADS1‐dependent delta‐5 desaturase (Δ5D) activity, respectively. ALA: α‐linolenic acid; LA: linoleic acid; GLA: γ‐linolenic acid; AA: arachidonic acid; EPA: eicosapentaenoic acid; DHA: docosahexaenoic acid. (F) Cell viability in TM4 cells was assessed 60 h after 10 µM SC26196 treatment in the presence of 4 µM GLA (FADS2‐derived fatty acid) and 4 µM DHA (fatty acid dependent on FADS2 activity). (G) Cell viability in hSCs was assessed after 48 h of treatment with 10 µM SC26196, with 16 µM GLA and 4 µM DHA added during the final 24 h. (H) ROS levels in TM4 cells treated with SC26196, GLA, and DHA. (I) Schematic of the TM4 spheroid formation in vitro. Red dots: fluorescent microplastics (Fluo‐MPs). (J,K) Representative immunofluorescence images showing penetration of Fluo‐MPs (red) into *Fads2*‐knockdown TM4 spheroids (J) and quantification of the relative area of Fluo‐MPs to DAPI (K). Scale bars indicate 100 µm. (L) Schematic of the human testicular tissue culture ex vivo. (M,N) Representative immunofluorescence images showing penetration of Fluo‐MPs (red) into seminiferous tubules of human testicular tissue after treatment with 20 µM SC26196 for 72 h (M) and quantification of Fluo‐MPs within seminiferous tubules (N). White dashed lines: seminiferous tubule boundaries; white arrows: Fluo‐MPs inside tubules; green arrows: Fluo‐MPs outside tubules. Scale bars indicate 100 µm. (O) Western blot analysis of proteins related to cellular junctions after *Fads2*‐knockdown or FADS2 inhibition in TM4. Occludin and Claudin‐11: integral membrane proteins of tight junctions; Vinculin: adaptor of tight junctions; β‐catenin: adaptor of gap junctions; E‐cadherin: integral membrane protein of anchoring junctions. (P,Q) Representative immunofluorescence images of Claudin‐11 (green) in *Fads2*‐knockdown TM4 spheroids (P) and quantification of fluorescence intensity (Q). Scale bars indicate 100 µm. (R,S) Representative images of Claudin‐11 (green) and DAPI (blue) in human testicular tissues between control and iNOA groups (R) and quantification of fluorescence intensity (S). white asterisk: inner seminiferous tubule; white arrows: Claudin‐11 (green). Scale bars indicate 100 µm. Data are expressed as mean ± SD of three independent experiments. *P* values were determined by Student's *t*‐test in (A, C, D, K, N, Q and S) and one‐way ANOVA in (B, F, G and H). ^*^ *p* < 0.05, ^**^ *p* < 0.01, ^***^ *p* < 0.001, n.s, not significant.

As a rate‐limiting desaturase, FADS2 governs the biosynthesis of n‐3 and n‐6 PUFAs, including metabolites such as GLA and DHA (Figure 2E). To test whether the depletion of these downstream products drives the loss of viability, we performed nutrient reconstitution experiments. Supplementation with exogenous GLA (a direct FADS2 product), but not ALA (a dietary precursor), partially rescued the viability defect caused by FADS2 inhibition (Figure S4E). Moreover, combined supplementation with GLA and DHA provided a superior rescue effect compared to GLA alone (Figure 2F and Figure S4F). Consistently, in hSCs treated with SC26196, combined GLA and DHA supplementation also restored cell viability (Figure 2G). Combined GLA and DHA supplementation also mitigated ROS levels in TM4 cells (Figure 2H). These findings support the essential role of FADS2‐derived PUFAs in maintaining Sertoli cell survival and redox homeostasis.

Given the critical role of Sertoli cells in the blood‐testis barrier [27], we next assessed barrier integrity in a TM4 spheroid model using fluorescent microplastics (Fluo‐MPs) as tracers (Figure 2I). Both *Fads2* knockdown and pharmacological FADS2 inhibition significantly increased Fluo‐MPs penetration into TM4 spheroids compared with controls (Figure 2J,K and Figure S4G,H). Notably, in an ex vivo human testicular tissue culture model (Figure 2L), FADS2 inhibition similarly increased the infiltration of Fluo‐MPs into seminiferous tubules (Figure 2M,N). Mechanistically, the tight‐junction protein Claudin‐11 was markedly downregulated, whereas other junctional proteins remained largely unaltered (Figure 2O and Figure S4I,J). This specific reduction in Claudin‐11 was recapitulated in the TM4 3D spheroid model (Figure 2P,Q) and, clinically, in testes from iNOA patients (Figure 2R,S). Thus, loss of FADS2 function compromises Sertoli cell viability and barrier integrity.

### 2.3 Loss of FADS2 Function Activates the STING Signaling Pathway

To elucidate the molecular mechanisms linking FADS2 deficiency to Sertoli cell dysfunction, we performed integrated RNA‐seq and proteomic profiling following *Fads2* knockdown, focusing on the 699 overlapping differentially expressed genes or proteins (DEGs or DAPs) (Figure S5A). Enrichment analysis of these overlapping features revealed a significant enrichment of immune and cellular stress‐related pathways, including “response to virus”, “response to interferon‐beta”, and “regulation of interferon‐beta production” (Figure 3A), suggesting the activation of a type I interferon program, a canonical downstream feature of stimulator of interferon genes (STING) signaling. We next examined whether loss of FADS2 function was associated with STING‐TBK1 signaling activation. Before assessing signaling responses, baseline comparison confirmed that FADS2 and STING proteins were detectable in both TM4 cells and hSCs (Figure S3B). Western blot analysis showed elevated phosphorylation of TBK1 and STING in *Fads2*‐knockdown TM4 cells (Figure S5B). Consistently, pharmacological inhibition of FADS2 increased p‐TBK1 and p‐STING levels in both TM4 cells and hSCs (Figure 3B and Figure S5C). Immunofluorescence revealed that FADS2 and STING predominantly colocalized in the cytoplasm under basal conditions, whereas FADS2 inhibition significantly disrupted this colocalization pattern (Figure 3C,D).

**FIGURE 3:**
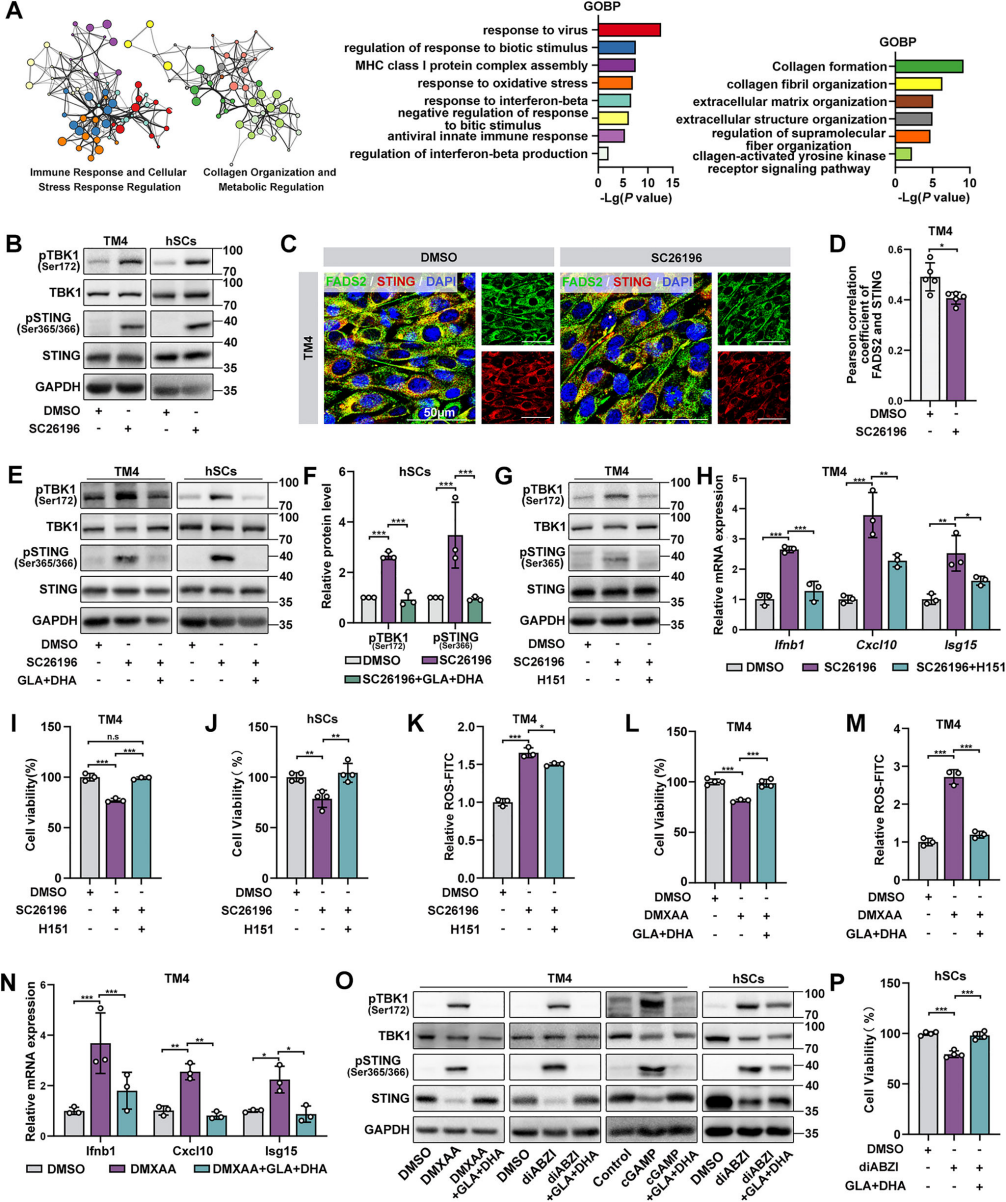
Loss of FADS2 function triggers STING activation in Sertoli cells. (A) Gene Ontology Biological Process (GOBP) enrichment analysis on the intersection of differentially expressed genes (DEGs) from transcriptomics and differentially abundant proteins (DAPs) from proteomics. Functional enrichment network of GOBP (left) and bar plots of representative significantly enriched GOBP terms. Significance thresholds: Fold change (FC) > 1.2 or < 0.833 and *p* < 0.05. (B) Western blot analysis of TBK1, p‐TBK1 (Ser172), STING, and p‐STING (Ser365 in mouse cells and Ser366 in human cells) in TM4 cells and hSCs following treatment with SC26196. (C,D) Representative immunofluorescence images of TM4 treated with SC26196 staining FADS2 (green) and STING (red) (C). Pearson's correlation coefficient values for co‐localization of FADS2 and STING in TM4 cells (D). Scale bars indicate 50 µm. (E) Western blot analysis of TBK1, p‐TBK1 (Ser172), STING, and p‐STING (Ser365/366) in TM4 cells and hSCs following SC26196 treatment with or without GLA and DHA supplementation. (F) Relative quantification of the hSCs Western blot bands in E. (G) Western blot of TBK1, p‐TBK1 (Ser172), STING, and p‐STING (Ser365) in TM4 cells treated with 10 µM SC26196 and 1 µM H‐151 (inhibitor of STING) for 60 h. (H) *Ifnb1*, *Isg15*, and *Cxcl10* mRNA levels in TM4 following treatment with SC26196 and H‐151. (I,J) Cell viability in TM4 cells (I) and hSCs (J) following treatment with SC26196 and H‐151. (K) ROS levels in TM4 cells following treatment with SC26196 and H‐151. (L,M) Cell viability (L) and ROS level (M) in TM4 cells stimulated with DMXAA in the presence of GLA and DHA. (N) *Ifnb1*, *Isg15*, and *Cxcl10* mRNA levels in TM4 cells stimulated with DMXAA in the presence of GLA and DHA. (O) Western blot analysis of TBK1, p‐TBK1 (Ser172), STING and p‐STING (Ser365/366) in TM4 cells treated with DMXAA, diABZI, 2’3’‐cGAMP or GLA and DHA, and in hSCs treated with diABZI or GLA and DHA. (P) Cell viability in hSCs stimulated with diABZI in the presence of 16 µM GLA and 4 µM DHA. Data are expressed as mean ± SD of three independent experiments. *p*‐values were determined by Student's *t*‐test in D and one‐way ANOVA in (F, H‐N and P). ^*^ *p* < 0.05, ^**^ *p* < 0.01, ^***^ *p* < 0.001, n.s, not significant.

Further examining the relationship between FADS2‐related metabolites and STING‐TBK1 signaling, we found that low‐dose GLA suppressed SC26196‐induced STING phosphorylation more effectively than ALA (Figure S5D). Based on this observation, combined supplementation with GLA and DHA reduced SC26196‐induced p‐TBK1 and p‐STING levels in both TM4 cells and hSCs (Figure 3E,F and Figure S5E). Consistently, supplementation with GLA and DHA partially restored FADS2‐STING colocalization in SC26196‐treated TM4 cells (Figure S5F,G). To clarify whether upstream cGAS sensing contributed to this response, RU.521 was used together with SC26196. RU.521 partially reduced SC26196‐induced p‐TBK1 and p‐STING levels, whereas GLA and DHA further suppressed these signals in the presence of RU.521, suggesting that cGAS contributes to, but does not fully account for, SC26196‐induced STING‐TBK1 activation (Figure S5H).

To determine if STING signaling functionally contributes to SC26196‐induced cellular stress, we treated TM4 cells with the STING inhibitor H‐151 in the presence of SC26196. Co‐treatment with H‐151 partially reduced SC26196‐induced p‐TBK1 and p‐STING levels (Figure 3G and Figure S5I) and restored FADS2‐STING colocalization (Figure S5J). Furthermore, H‐151 downregulated the expression of *Ifnb1* and interferon‐stimulated genes (ISGs: *Isg15* and *Cxcl10*) in TM4 cells (Figure 3H), partially rescued cell viability in TM4 cells and hSCs (Figure 3I,J), and rescued the ROS levels caused by SC26196 in TM4 cells (Figure 3K). Conversely, direct activation of STING with the agonist DMXAA mirrored several effects of FADS2 inhibition, decreasing viability and increasing ROS level (Figure S6A,B). We then verified whether FADS2‐derived PUFAs modulate this pathway. Co‐treatment with GLA and DHA reduced DMXAA‐induced p‐STING and p‐TBK1 levels, suppressed *Ifnb1*, *Isg15*, and *Cxcl10* expression, improved cell viability, reduced ROS levels, and partially restored FADS2‐STING colocalization (Figure 3L–O and Figure S6C,D). Furthermore, GLA and DHA also reduced diABZI‐ and 2’3’‐cGAMP ‐induced p‐STING and p‐TBK1 levels in TM4 cells and attenuated diABZI‐induced p‐STING activation and viability loss in hSCs (Figure 3O,P and Figure S6D).

To further evaluate the in vivo relevance of pharmacological FADS2 inhibition, mice were administered SC26196 with or without ALA or combined GLA/DHA supplementation (Figure S7A). SC26196 reduced sperm density and motility, disrupted seminiferous tubule architecture, and increased testicular p‐TBK1 and p‐STING levels (Figure S7B–E). These effects were partially attenuated by PUFA supplementation. Notably, combined GLA and DHA supplementation showed more pronounced effects than ALA in restoring sperm concentration and suppressing testicular STING‐TBK1 activation in the context of pharmacological FADS2 inhibition (Figure S7B–E). Collectively, these data indicate that FADS2 inhibition is associated with STING‐TBK1 activation and spermatogenic injury, which can be attenuated by PUFA supplementation, particularly GLA and DHA.

### 2.4 Sertoli Cell‐Derived IFN‐β Primes Germ Cells for Heat Shock‐Induced Necroptosis

STING activation is known to drive type I interferon production [28, 29]. Consistent with this, ELISA of TM4 cell culture supernatants showed that DMXAA stimulation, as well as *Fads2* knockdown or FADS2 inhibition, markedly increased IFN‐β secretion (Figure 4A). This effect was recapitulated in hSCs and the ex vivo human testicular tissue culture model (Figure 4B,C). Since heat shock (HS)‐induced necroptosis involves IFN‐β‐driven ZBP1 signaling [30], we hypothesized that Sertoli cell‐derived IFN‐β might sensitize germ cells to stress‐induced death. GC‐1spg and GC‐2spd germ cells were pretreated with recombinant IFN‐β followed by HS, and necroptosis was assessed by MLKL phosphorylation (p‐MLKL) (Figure 4D). In GC‐1spg cells, IFN‐β treatment across the tested concentration range (1–20 ng/mL) enhanced HS‐induced p‐MLKL levels (Figure 4E). In GC‐2spd cells, higher doses (5–20 ng/mL) were required to elicit a clear increase in p‐MLKL (Figure 4F).

**FIGURE 4:**
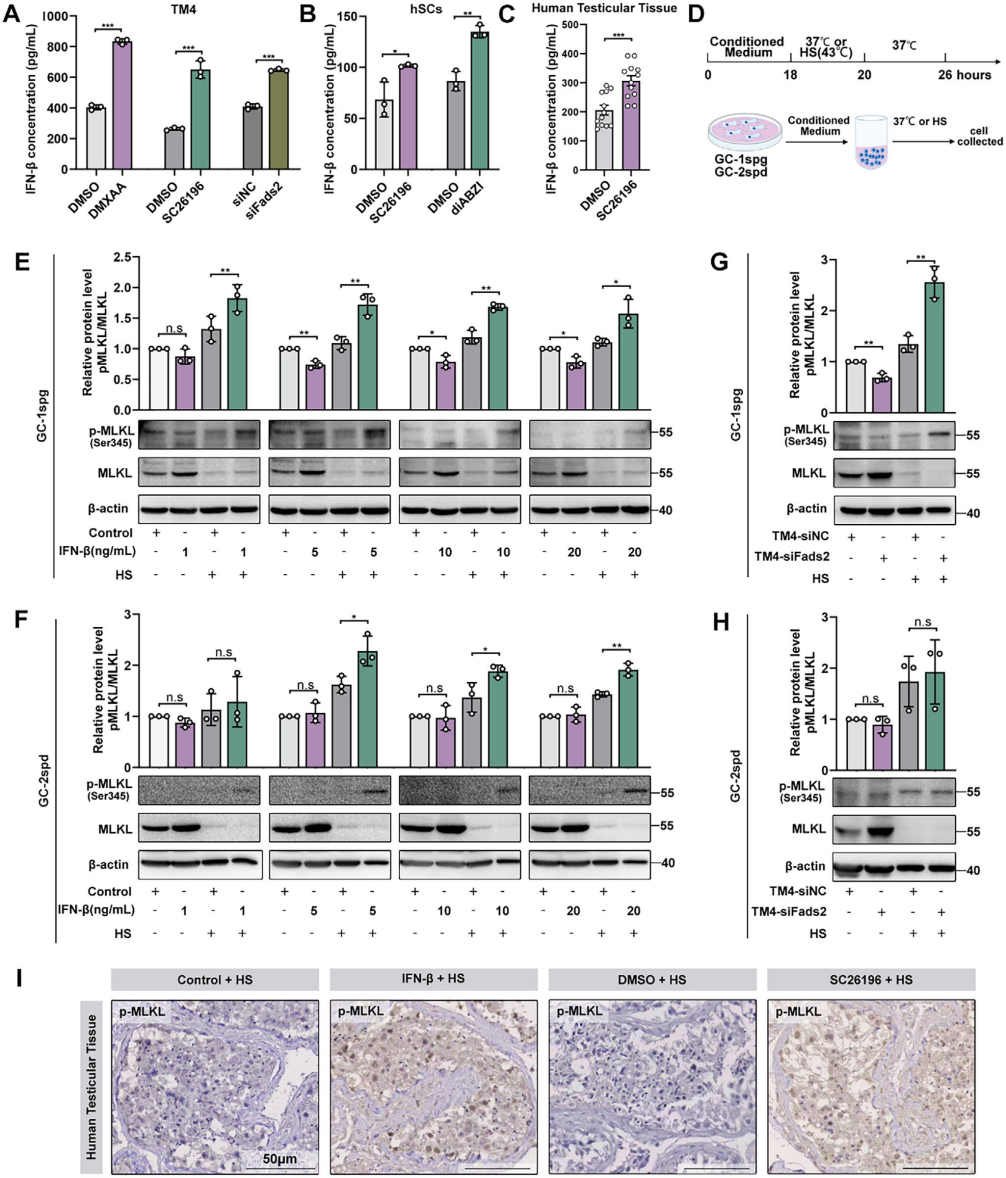
Sertoli cell‐derived IFN‐β primes germ cells for heat shock‐induced necroptosis. (A) IFN‐β levels in TM4 cell culture supernatants after DMXAA stimulation, *Fads2* knockdown, or FADS2 inhibition. (B) IFN‐β levels in hSCs culture supernatants following treatment with SC26196 and diABZI. (C) IFN‐β levels in the culture supernatants of human testicular tissue treated with SC26196. (D) Schematic of the experimental design for heat shock (HS) in GC‐1spg and GC‐2spd. (E,F) Western blot analysis and quantification of MLKL and p‐MLKL (Ser345) in GC‐1spg (E) and GC‐2spd (F), treated with IFN‐β followed by HS. (G,H) Western blot analysis and quantification of MLKL and p‐MLKL (Ser345) in GC‐1spg (G) and GC‐2spd (H), exposed to culture supernatants from TM4 cells after knockdown of *Fads2* for 60 h, followed by HS. (I) Representative Immunohistochemistry images of human testicular tissues treated with 20 ng/mL IFN‐β or 20 µM SC26196 for 48 h and subsequently subjected to HS. Scale bars indicate 50 µm. Data in (A and B) are expressed as mean ± SD of three independent experiments. Data in C are expressed as mean ± SEM. *P* values were determined by Student's *t*‐test in (A, B, C, and E–H). ^*^ *p* < 0.05, ^**^ *p* < 0.01, ^***^ *p* < 0.001, n.s, not significant.

To test whether IFN‐β released from *Fads2*‐knockdown TM4 cells can promote germ‐cell necroptosis, we exposed GC‐1spg and GC‐2spd cells to conditioned media from *Fads2*‐knockdown TM4 cells, followed by HS. Under these conditions, HS markedly increased p‐MLKL in GC‐1spg cells (Figure 4G), whereas GC‐2spd cells showed no obvious change (Figure 4H). Finally, in an ex vivo human testicular tissue culture model, combined treatment with IFN‐β or SC26196 and HS also led to an increase in p‐MLKL within seminiferous tubules (Figure 4I). Together, these data support a model in which FADS2‐STING‐driven IFN‐β production in Sertoli cells primes germ cells and testicular tissue for HS‐induced necroptosis.

### 2.5 FADS2‐STING Signaling Drives Pro‐Fibrotic Transcriptional Reprogramming and Peritubular Fibrosis

Given that STING activation is implicated in tissue fibrosis [31], we investigated the link between FADS2‐STING signaling and collagen deposition. We performed RNA‐seq on DMXAA‐activated TM4 cells (Figure S9A) and analyzed 333 overlapping differentially expressed genes or proteins (DEGs or DAPs) (Figure 5A). Enrichment analysis revealed significant upregulation of collagen fibril organization alongside immune pathways (Figure 5B). Heatmap analysis confirmed that STING activation, similar to *Fads2* knockdown, induced a broad upregulation of collagen genes, indicative of collagen reprogramming (Figure S9B–D). To assess tissue‐level structural remodeling, we applied stimulated Raman scattering (SRS) and second harmonic generation (SHG) imaging [32] to human testicular tissue sections (Figure S8) and quantified collagen thickness based on SHG signals, with SRS signals as tissue morphological references (Figure 5C). In the ex vivo human testicular tissue culture model, SC26196 treatment increased SHG signal intensity, revealing localized collagen thickening along the seminiferous tubule basement membrane (STBM) (Figure 5D,E), a feature characteristic of peritubular fibrosis [33]. Western blot analysis in TM4 cells showed consistent upregulation of COL1A1 and COL1A2 upon FADS2 inhibition, whereas COL3A1 showed only a slight upward trend (Figure 5F). Supporting this observation, direct activation of STING with DMXAA, diABZI, or 2’3’‐cGAMP also increased COL1A1 and COL1A2 expression in TM4 cells (Figure S9E). Similar induction of COL1A1 and COL1A2 was observed in hSCs following SC26196 or diABZI treatment (Figure S9E). Consistently, immunofluorescence and immunohistochemistry in cultured human testicular tissue show increased COL1A1 deposition along the STBM following SC26196 treatment (Figure 5G,H).

**FIGURE 5:**
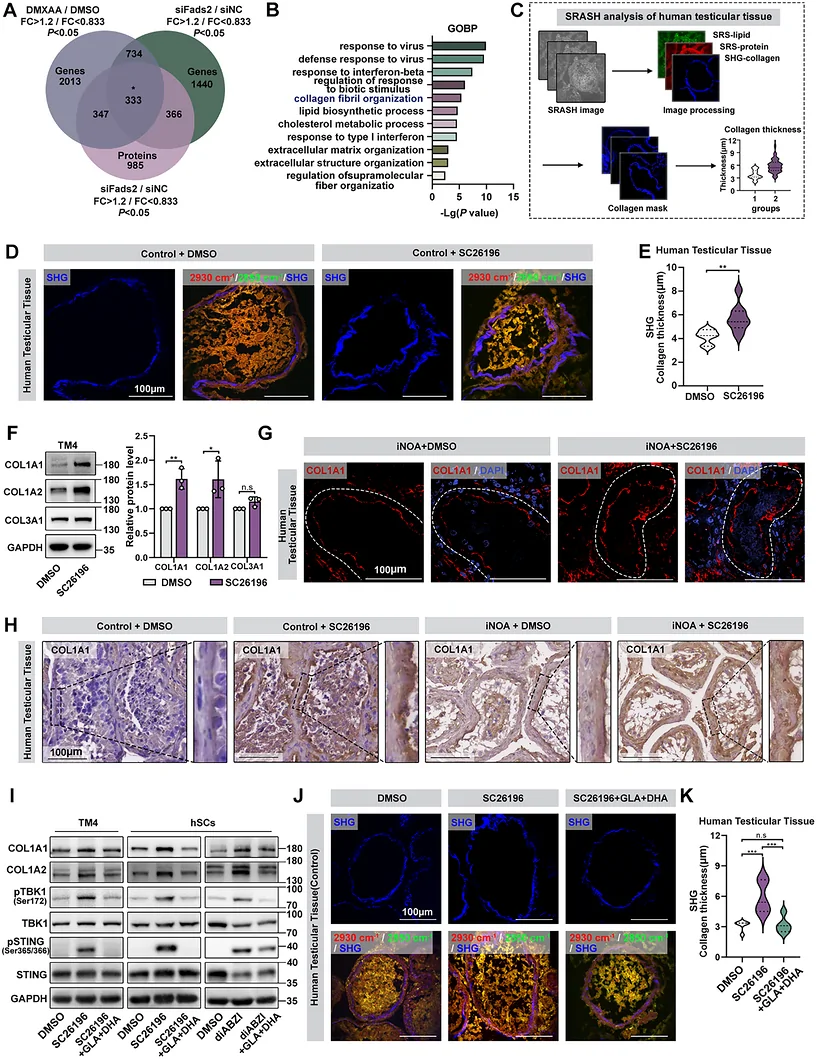
FADS2‐STING signaling drives pro‐fibrotic transcriptional reprogramming and peritubular collagen deposition. (A) Venn diagram showing the overlap between differentially expressed genes (DEGs) and differentially abundant proteins (DAPs). Significance thresholds: FC > 1.2 or < 0.833 and *p* < 0.05. (B) Enrichment analysis on the intersection of DEGs and DAPs from TM4 transcriptomics (si*Fads2* vs. siNC and DMXAA vs. DMSO) and TM4 proteomics (si*Fads2* vs. siNC). (C) Schematic of the experimental workflow for measuring collagen thickness based on SHG signal in human testicular seminiferous tubules using SRASH imaging. (D,E) SRASH images of seminiferous tubules of human testicular tissue ex vivo treated with SC26196 (D) and quantitative analysis of the collagen thickness based on SHG signal (E), *n* = 5. SHG (blue): collagen; 2850cm^−1^ (green): lipid; 2930cm^−1^ (red): protein. Scale bars indicate 100 µm. (F) Western blot analysis and quantification of COL1A1, COL1A2, and COL3A1 in TM4 cells after inhibition of FADS2. (G) Representative immunofluorescence images of COL1A1 in human testicular tissue ex vivo treated with SC26196. Scale bars indicate 100 µm. (H) Representative immunohistochemistry images of human testicular tissues ex vivo treated with SC26196. (I) Western blot of COL1A1 and COL1A2 in TM4 cells treated with SC26196, GLA, and DHA, or in hSCs treated with SC26196, diABZI, GLA, and DHA. (J,K) Representative SRASH images of seminiferous tubules of human testicular tissue ex vivo treated with SC26196 with supplementation of GLA and DHA (J). Quantitative analysis of the collagen thickness based on SHG signal, *n* = 5 (K). Scale bars indicate 100 µm. *P* values were determined by Student's *t*‐test in (E) and one‐way ANOVA in (F and K). ^**^ *p* < 0.01, ^***^ *p* < 0.001, n.s., not significant.

Importantly, we examined whether STING inhibition or FADS2‐related PUFA replenishment could attenuate collagen induction. The STING inhibitor H‐151 attenuated SC26196‐induced *Col1a1* and *Col1a2* upregulation at the mRNA level in TM4 cells (Figure S9F). GLA and DHA supplementation also downregulated *Col1a1* and *Col1a2* transcripts and proteins in TM4 cells treated with SC26196 (Figure 5I and Figure S9G,H). Further protein‐level validation showed that GLA and DHA also reduced COL1A1 and COL1A2 expression induced by direct STING agonists, including DMXAA, diABZI, and 2’3’‐cGAMP in TM4 cells (Figure S9I,J). Consistently, GLA and DHA supplementation reduced SC26196‐ or diABZI‐induced COL1A1 and COL1A2 expression in hSCs (Figure 5I and Figure S9G). Similar reductions in *COL1A1* and *COL1A2* transcripts were observed in ex vivo human testicular tissue treated with SC26196 in combination with H‐151 or with GLA and DHA (Figure S9K), and COL1A1 immunohistochemistry confirmed decreased type I collagen staining upon supplementation with GLA and DHA (Figure S9L). Consistently, co‐treatment with GLA and DHA attenuated the STBM collagen thickening as quantified by SHG imaging (Figure 5J,K). These findings suggest that FADS2 deficiency triggers structural remodeling of the human testis through STING‐dependent collagen deposition.

### 2.6 Peritubular Fibrosis Correlates With Reduced FADS2 Expression and Poor Sperm Retrieval Outcomes

We next interrogated the clinical relevance of these findings. SRS and SHG imaging of iNOA patient testes revealed a markedly increased SHG signal and localized collagen thickening along the STBM compared with controls, consistent with H&E observations of STBM thickening, which indicated peritubular fibrosis (Figure 6A,B). Quantitative analysis showed a significant inverse correlation between FADS2 protein intensity and peritubular collagen thickness (Figure 6C). At the single‐cell level, scRNA‐seq analysis confirmed that Sertoli cells from iNOA testes exhibited specific upregulation of multiple collagen genes, including COL1A1 and COL1A2 (Figure 6D).

**FIGURE 6:**
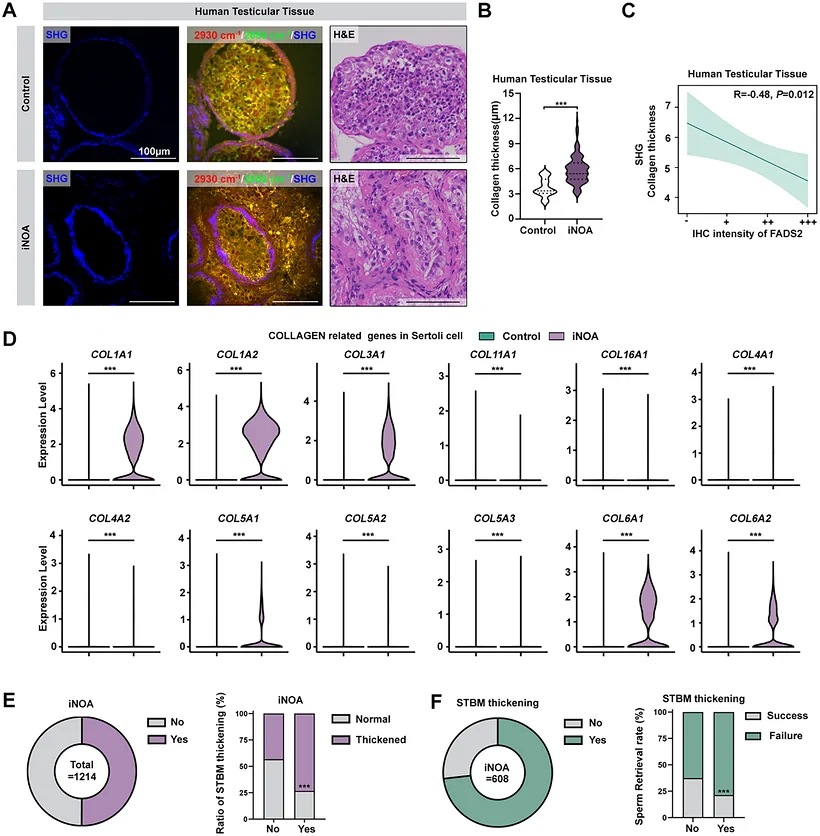
Peritubular fibrosis correlates with reduced FADS2 expression and poor sperm retrieval outcomes in iNOA patients. (A,B) Representative SRASH and hematoxylin and eosin (H&E) staining images of seminiferous tubules of human testes from Control (*n* = 6) and iNOA (*n* = 20) (A). Quantitative analysis of the collagen thickness based on SHG signal (B). Scale bars indicate 100 µm. (C) The Spearman correlation between IHC‐based FADS2 expression intensity within seminiferous tubules and the SHG signal‐based collagen thickness in testicular tissue. (D) Violin plot showing expression of COLLAGEN‐related genes in Sertoli cells from scRNA‐seq datasets GSE149512 and GSE112013. (E) The etiologic distribution of male infertility at a single center and the percentage of patients whose testicular histology showed thickening of the seminiferous tubule wall. (F) Proportion of STBM thickening in the iNOA population and sperm retrieval success rates between iNOA patients with STBM thickening and those without. STBM: seminiferous tubule basement membrane. *p*‐values in (B) were determined by Student's *t*‐test and (F) were determined by χ^2^ test. ^***^ *p* < 0.001.

To evaluate the clinical relevance of STBM thickening, we analyzed etiologic patterns and sperm retrieval outcomes in our iNOA cohort. iNOA patients exhibited a significantly higher prevalence of histological STBM thickening (Figure 6E). Crucially, within the iNOA cohort, patients with thickened STBM had a significantly lower sperm retrieval success rate than those without STBM thickening (21.35% vs. 37.42%, *p* < 0.001, Figure 6F and Table S1). Baseline clinical characteristics were generally comparable between the two groups, except for a modest difference in E_2_ levels (Table S1). To account for potential confounding, we further performed logistic regression analysis. STBM thickening was associated with lower odds of successful sperm retrieval in the crude model (OR = 0.454, 95% CI: 0.307‐0.670, *p* < 0.001), after adjustment for age and BMI (OR = 0.465, 95% CI: 0.312, *p* < 0.001) and after further adjustment for age, BMI, E_2_, FSH, LH, PRL, and T (OR = 0.482, 95% CI: 0.313‐0.743, *p* = 0.001; Table S2). Together, these findings indicate that STBM thickening is independently associated with reduced odds of successful sperm retrieval in iNOA, supporting peritubular collagen deposition as a clinically relevant histological correlate of impaired spermatogenic potential.

### 2.7 Dietary GLA and DHA Mitigation of Busulfan‐Induced Spermatogenic Injury and Collagen Remodeling In Vivo

To evaluate the therapeutic potential of FADS2 downstream PUFAs under spermatogenic injury conditions, we utilized a low‐dose busulfan‐induced spermatogenic injury model (Figure 7A). Busulfan treatment markedly reduced testis size and weight, whereas oral supplementation with GLA and DHA partially restored testis morphology and significantly increased the testicular index (Figure 7B,C). Epididymal sperm analysis showed that GLA and DHA co‐treatment improved sperm concentration and sperm motility (Figure 7D,E). In fertility tests, supplemented males yielded more pregnant females and a greater total number of offspring compared to the vehicle‐treated busulfan group (Figure 7F,G). Histologically, while busulfan caused severe tubular atrophy, GLA and DHA supplementation preserved seminiferous tubule structure and increased the proportion of tubules exhibiting complete spermatogenesis (Figure 7H,I).

**FIGURE 7:**
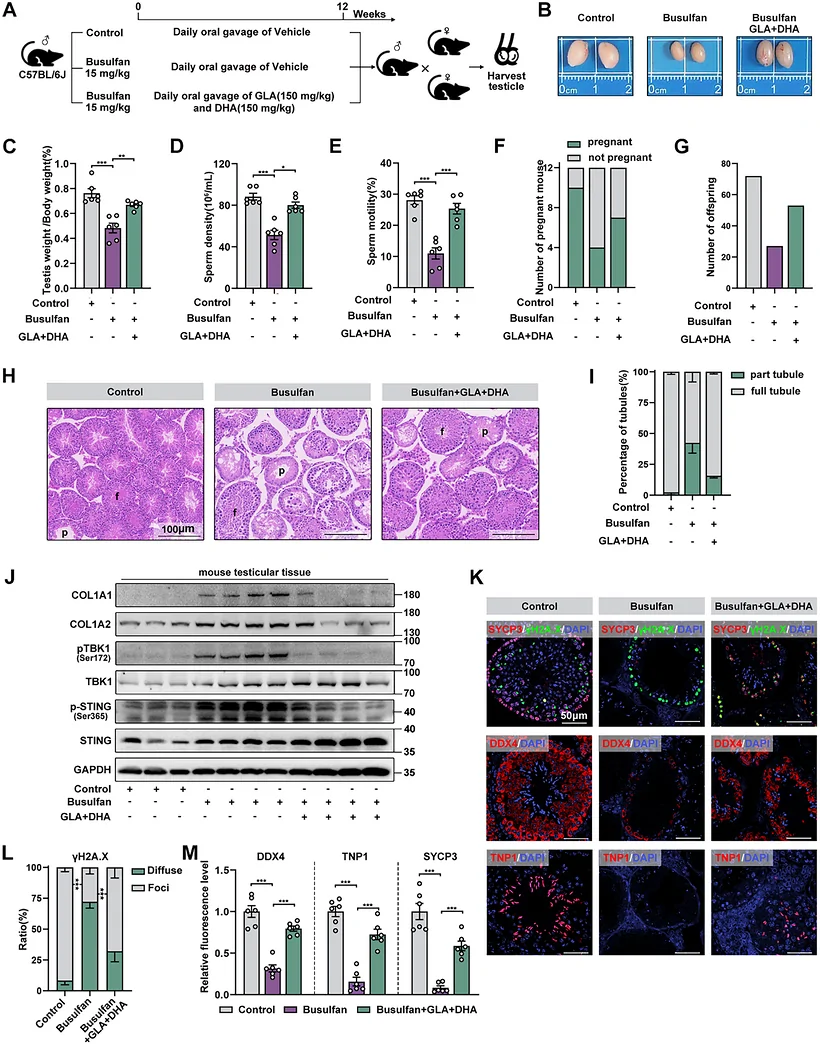
Dietary GLA and DHA supplementation mitigates testicular fibrosis and partially restores spermatogenesis in a busulfan‐induced mouse model. (A) Schematic diagram of the animal experiments. (B) Representative images of testis gross morphology. (C) Comparison of testicular organ index (testis weight/body weight × 100%) among groups (*n* = 6 per group). (D,E) Sperm density (million/mL) (D) and sperm motility (%) (E) between groups. (F,G) Number of pregnant females (F) and number of offspring (G) after mating with males from each group. (H) Representative hematoxylin and eosin (H&E) staining images across groups. (I) Quantification of seminiferous tubules exhibiting partial or full spermatogenesis based on H&E staining images. p: part tubule; f: full tubule. Scale bars indicate 100 µm. (J) Western blot of TBK1, p‐TBK1 (Ser172), STING, p‐STING (Ser365), COL1A1, and COL1A2 in mouse testicular tissue from each group. (K–M) Representative immunofluorescence images staining for DDX4 (marker of germ cells), TNP1 (marker of spermatids), SYCP3 (marker of spermatocytes), and γH2A.X (marker of cells with DNA double‐strand breaks) in mouse testicular sections (K). Percentage of nuclei with different γH2A.X staining patterns (L) and Quantification of fluorescence intensity (M). Scale bars indicate 50 µm. Data are represented as mean ± SEM. *P* values in (C, D, E, L, and M) were determined by one‐way ANOVA. ^*^ *p* < 0.05, ^**^ *p* < 0.01, ^***^ *p* < 0.001.

Mechanistically, consistent with in vitro findings, busulfan increased testicular p‐TBK1 and p‐STING levels, accompanied by elevated COL1A1 and COL1A2 expression. In contrast, GLA and DHA supplementation suppressed p‐STING and p‐TBK1 levels and concurrently attenuated collagen accumulation (Figure 7J). To assess the impact of GLA and DHA on germ cells, we analyzed the DNA damage marker γH2A.X and spermatogenic cell markers, including SYCP3 (spermatocytes), DDX4 (germ cells) and TNP1 (spermatids). Busulfan‐treated testes showed diffuse, pan‐nuclear γH2A.X staining indicative of widespread DNA damage and a loss of germ cells expressing SYCP3, DDX4, or TNP1 (Figure 7K). Conversely, GLA and DHA treatment restored the discrete γH2A.X foci pattern characteristic of meiotic recombination and significantly preserved spermatocyte and spermatid populations (Figure 7L,M). These findings indicate that dietary GLA and DHA supplementation attenuates busulfan‐induced spermatogenic injury, STING‐TBK1 activation, and collagen‐related remodeling in vivo.

Because GLA and DHA supplementation reduced busulfan‐induced STING‐TBK1 activation, we further asked whether STING signaling functionally contributes to busulfan‐induced spermatogenic injury. In a parallel experiment, wild‐type (WT) and *Sting‐*knockout (KO) mice were treated with busulfan (Figure S10A). Compared with the busulfan‐treated WT group, the STING‐deficient group showed significantly higher sperm density and a non‐significant trend toward improved sperm motility (Figure S10B,C). Histological analysis further indicated partial preservation of seminiferous tubule architecture and less prominent collagen accumulation along STBM in STING‐deficient mice after busulfan exposure (Figure S10D,E). Western blot analysis showed reduced STING‐TBK1 activation in STING‐deficient testes under busulfan treatment (Figure S10F). These findings support a functional contribution of STING signaling to busulfan‐induced testicular injury. Notably, testicular FADS2 protein levels and p‐MLKL levels were not markedly altered in busulfan‐treated mice (Figure S10G). Together, these in vivo data indicate that GLA and DHA supplementation attenuates busulfan‐induced spermatogenic injury and collagen‐related remodeling, while STING deficiency provides genetic support for the contribution of STING signaling to this injury context.

## 3 Discussion

In this study, we define the pathological relevance of Sertoli cell‐associated FADS2‐PUFA remodeling in iNOA and link reduced FADS2 expression to STING‐TBK1 activation, increased germ‐cell vulnerability, and peritubular collagen remodeling. These findings extend the prevailing germ cell‐centered view of spermatogenic failure by highlighting Sertoli cells as metabolically responsive regulators of the spermatogenic niche, with clinical relevance supported by the association between STBM thickening and reduced sperm retrieval success.

Dysregulated unsaturated fatty acid metabolism is increasingly recognized as a driver and amplifier of diverse metabolic, inflammatory, and fibrotic diseases [34]. In male infertility, alterations in seminal plasma fatty‐acid composition have been documented in NOA patients [35, 36], and transcriptomic profiling together with MALDI imaging has indicated reduced unsaturated lipids in NOA testes [15, 37], although the underlying mechanisms remain incompletely defined. Focusing on iNOA specifically, the specific subtype of NOA, we observed a shift in testicular lipid composition from PUFA‐enriched species toward more saturated phospholipids. By integrating single‐cell analyses, we further pinpointed a selective reduction of the PUFA desaturase FADS2 in Sertoli cells, providing an enzymatic anchor to explain this lipid shift. Functionally, genetic depletion or pharmacologic inhibition of FADS2 compromised Sertoli‐cell barrier properties, at least in part via downregulation of the tight‐junction component Claudin‐11, consistent with genetic evidence that FADS2 loss disrupts BTB integrity and spermatogenesis in mice [23]. Nevertheless, the precise upstream and downstream mechanisms by which impaired FADS2‐dependent desaturation perturbs Sertoli‐cell homeostasis and ultimately precipitates spermatogenic failure in iNOA warrant further investigation.

Sertoli cells are not only structural components of the BTB but also central regulators of the testicular immune microenvironment [38, 39]. Under physiological conditions, Sertoli cells sustain an immune‐privileged but infection‐aware milieu through coordinated secretion of immunomodulatory factors and growth signals [40, 41]. In contrast, inflammatory or autoimmune conditions reprogram the Sertoli cell secretome toward higher levels of proinflammatory cytokines, chemokines, and profibrotic mediators, thereby promoting BTB disruption, germ‐cell injury, and fibrotic remodeling [42]. In this setting, we found that *Fads2* knockdown or pharmacological FADS2 inhibition activated STING‐TBK1 signaling in Sertoli‐cell models, accompanied by enhanced antiviral innate immune and type I interferon‐related responses. STING is a pivotal adaptor that triggers inflammatory programs largely through type I interferons [43]. Notably, prior work reported a physical interaction between STING and FADS2, and suggested that STING activation is accompanied by reduced spatial association between the two proteins, reflected by a decreased Pearson's colocalization [44]. Consistent with this concept, FADS2 knockdown or inhibition reduced FADS2‐STING colocalization in our Sertoli‐cell models, providing supportive evidence of STING engagement under conditions of impaired PUFA desaturation. PUFAs have long been studied as modulators of inflammation [45], classically through eicosanoid remodeling and suppression of NF‐κB‐linked cytokine programs [46, 47]. Here, we extend this framework in Sertoli cells by showing that FADS2‐derived PUFAs, particularly GLA and DHA, attenuate STING‐TBK1 activation. Although the cGAS inhibitor RU.521 partially reduced SC26196‐induced STING‐TBK1 activation, GLA and DHA further attenuated this response in the presence of RU.521, suggesting that cGAS‐dependent stress sensing contributes to, but does not fully account for, FADS2 inhibition‐associated STING‐TBK1 activation. However, the protective effects of GLA and DHA should not be attributed exclusively to STING inhibition, as PUFAs may also influence membrane composition, oxidative stress, and other inflammatory pathways. Contributions from STING‐independent mechanisms therefore cannot be excluded. Collectively, these results connect impaired FADS2‐dependent PUFA remodeling in Sertoli cells to STING‐TBK1‐associated innate immune activation in iNOA‐relevant contexts.

Downstream of STING activation, increased expression of IFN‐β leads to elevated expression of necroptosis‐associated mediators such as ZBP1 and MLKL, thereby lowering the threshold for necroptosis [48]. Importantly, STING‐dependent IFN production is necessary but not sufficient for necroptosis [49]. This aligns with a two‐hit framework in which type I interferon acts as a first hit that primes cells for necroptosis, whereas an environmental or inflammatory stressor provides the second hit that triggers overt necroptotic execution [50]. Consistent with this model, we found that low‐dose IFN‐β exposure, either by exogenous supplementation or conditioned medium from *Fads2*‐knockdown TM4 cells, rendered GC‐1spg more susceptible to necroptosis than GC‐2spd. This differential sensitivity provides a plausible mechanistic explanation for the preferential occurrence of necroptosis in spermatogonia reported in iNOA testes [30]. In vivo p‐MLKL analysis further indicated that busulfan treatment did not induce overt MLKL phosphorylation under the current experimental conditions. Because busulfan‐treated mice were not subjected to an additional stress challenge, such as HS, this finding is consistent with a priming model in which IFN‐β‐associated signaling increases germ‐cell susceptibility to necroptosis, whereas additional stress stimuli may be required for robust necroptosis execution.

Beyond inflammatory priming and stress‐sensitive germ‐cell loss, our data indicated that the Sertoli cell FADS2‐PUFA‐STING axis contributes to collagen‐related remodeling of the spermatogenic niche, characterized by collagen deposition and thickening of STBM. Integrated enrichment analysis of overlapping transcriptomic and proteomic changes in TM4 cells highlighted activation of extracellular matrix programs, including collagen fibril organization, together with coordinated upregulation of collagen‐related genes. In ex vivo human testicular cultures, pharmacologic inhibition of FADS2 increased SHG signal along the STBM, consistent with enhanced type I collagen deposition and aligned with prior reports describing elevated type I collagen in iNOA testes [51]. Notably, STBM thickening is a recognized histopathological feature of severe spermatogenic failure and iNOA, concordant with the increased SHG signal observed in our patient specimens [52, 53]. Under physiological conditions, Sertoli cells secrete Collagen IV and Laminin, which contribute to the formation of STBM [54]. In pathological inflammatory settings, the peritubular niche can shift toward a profibrotic state, characterized by increased deposition of fibrillar collagens and fibronectin, thereby promoting peritubular fibrosis [55]. Extending these concepts, our results provide metabolic‐immune evidence that FADS2 downregulation or inhibition, as well as STING activation, enhance collagen production, especially type I collagen, across murine and human Sertoli‐cell contexts. Importantly, both a STING inhibitor and supplementation with FADS2‐derived PUFAs (GLA and DHA) attenuated this profibrotic response, reducing collagen production and limiting collagen accumulation at the STBM, supporting a tractable link between metabolically gated STING‐TBK1 activation and peritubular fibrosis. Finally, the association between increased STBM thickness and reduced sperm retrieval success underscores the clinical relevance of this structural alteration and is consistent with prior observations linking peritubular fibrosis to testicular stiffness and impaired spermatogenic function [56].

As a proof‐of‐concept for in vivo tractability, we leveraged the busulfan‐induced testicular injury model, which captures key features of germ‐cell depletion and niche stress [57]. Dietary supplementation with GLA and DHA reduced testicular STING‐TBK1 activation and concurrently attenuated profibrotic readouts, while improving epididymal sperm parameters and preserving spermatogenic markers. Moreover, STING deficiency partially protected against busulfan‐induced injury, primarily reflected by improved sperm density, histological preservation, reduced collagen deposition, and lower TBK1 activation. These findings support a functional contribution of STING signaling to busulfan‐induced testicular injury and suggest that PUFA replenishment can mitigate STING‐associated spermatogenic niche damage in vivo.

This study defines the pathological relevance of Sertoli cell‐associated FADS2‐PUFA remodeling in iNOA by linking reduced FADS2 expression to STING‐TBK1 signaling, germ‐cell stress vulnerability, and collagen‐related STBM remodeling. Together with the association between STBM thickening and reduced sperm retrieval success, these findings support Sertoli cell lipid metabolism as a clinically relevant component of spermatogenic niche deterioration and provide a rationale for PUFA replenishment as a potential strategy to attenuate STING‐associated inflammatory activation and collagen remodeling in iNOA.

### 3.1 Limitations of the Study

This study provides pathological insights into iNOA by delineating a Sertoli cell‐associated metabolic‐innate immune framework linking FADS2‐mediated fatty acid remodeling to STING‐associated inflammatory activation and spermatogenic niche deterioration. However, several limitations warrant further consideration. First, although our data indicate that reduced FADS2 activity promotes collagen deposition at the STBM and that STBM thickness is negatively associated with sperm retrieval success, the biochemical and biophysical mechanisms by which STBM remodeling disrupts spermatogenesis remain to be fully elucidated. Second, although GLA and DHA attenuated STING‐TBK1 activation, the precise mechanism by which impaired FADS2 function leads to STING activation remains to be further investigated. Whether this process is mediated by altered membrane lipid composition, disrupted FADS2‐STING spatial organization, cGAS‐dependent stress sensing, or other upstream stress signals requires further investigation. Third, our findings suggest that STING activation reprograms Sertoli cells toward a proinflammatory and profibrotic secretory phenotype. Nevertheless, it is still unclear whether these secreted factors act directly on peritubular myoid cells or instead recruit additional immune cell types that cooperate to drive extracellular matrix accumulation and STBM thickening. Fourth, although the busulfan model supports a functional contribution of STING signaling to spermatogenic injury and collagen deposition in vivo, it cannot directly recapitulate the Sertoli cell‐associated FADS2‐PUFA remodeling abnormalities observed in human iNOA testes. Therefore, this model should be interpreted as an in vivo proof‐of‐concept model for evaluating spermatogenic injury, collagen remodeling, and the contribution of STING signaling in a chemically induced injury context, rather than as a direct model of the FADS2 deficiency‐driven lipid metabolic etiology of iNOA. Finally, the clinical association between STBM thickness and sperm retrieval success was assessed retrospectively, and residual confounding by unmeasured clinical factors cannot be excluded despite adjustment for measured covariates in multivariable logistic regression analyses. Larger multi‐center clinical cohorts will be required to confirm the robustness of the relationships among FADS2 expression, STBM collagen deposition, and sperm retrieval success across diverse iNOA subpopulations.

## 4 Conclusion

In summary, this study defines the pathological relevance of Sertoli cell‐associated FADS2‐PUFA remodeling to innate immune activation and spermatogenic niche deterioration in iNOA. By integrating human testicular lipidomics with single‐cell transcriptomic analyses, we found that iNOA is characterized by a shift from PUFA‐enriched toward more saturated phospholipids, accompanied by a selective reduction of the PUFA desaturase FADS2 in Sertoli cells. Mechanistically, FADS2 deficiency was linked to STING‐TBK1 activation and a type I interferon program, which sensitizes germ cells to stress‐triggered necroptosis and promotes collagen I accumulation and thickening of the seminiferous tubule basement membrane. Importantly, replenishment with FADS2‐derived PUFAs (GLA and DHA) or pharmacologic STING inhibition mitigates these inflammatory and profibrotic responses. And GLA and DHA supplementation partially improved spermatogenic outcomes, while STING deficiency provided genetic evidence for a contribution of STING to busulfan‐associated testicular injury. Collectively, our findings define the pathological relevance of Sertoli cell‐associated FADS2‐PUFA remodeling in iNOA and support PUFA replenishment as a potential strategy to attenuate STING‐associated inflammatory activation and collagen remodeling within the spermatogenic niche.

## 5 Experimental Section

### 5.1 Human Testicular Tissue Collection and Preservation

Human testicular tissues were obtained from patients who underwent microsurgical testicular sperm extraction (micro‐TESE) or diagnostic testicular biopsy at the Reproductive Medicine Center of the Third Affiliated Hospital of Guangzhou Medical University. Based on postoperative histopathology and clinical data, patients were classified into a control group (obstructive azoospermia or ejaculatory dysfunction) and an idiopathic nonobstructive azoospermia (iNOA) group. All patients provided written informed consent prior to inclusion. The study was conducted in accordance with the Declaration of Helsinki and was approved by the Ethics Committee of the Third Affiliated Hospital of Guangzhou Medical University (Approval No. 2025148). Fresh testicular tissue obtained during surgery was immediately placed in ice‐cold PBS and transported to the laboratory. The samples were subjected to different treatments for various experiments: (1) Snap‐frozen in liquid nitrogen for lipidomics, protein, and RNA extraction; (2) Embedded in optimal cutting temperature (OCT) compound and snap‐frozen for subsequent immunohistochemistry (IHC); (3) Maintained under appropriate conditions for in vitro human testicular tissue culture and functional assays.

### 5.2 Lipidomics Analysis

Lipidomic profiling of human testicular tissue was performed by Beijing Novogene Co., Ltd. Human testicular specimens were placed into pre‐chilled homogenization tubes and extracted with an organic solvent system. After homogenization and centrifugation, the organic phase was collected. The supernatants were combined, evaporated to dryness under a gentle stream of nitrogen, and reconstituted in organic solvent for liquid chromatography‐tandem mass spectrometry (LC‐MS/MS) analysis. Full‐scan mass spectra were acquired in both positive and negative ion modes. The raw mass spectrometry files were imported into Compound Discoverer 3.1 software for spectral processing and database searching, and the resulting data were used for subsequent analyses.

### 5.3 Integration and Analysis of Public Single‐Cell RNA‐seq Datasets

All public datasets were obtained from the Gene Expression Omnibus (GEO) database (https://www.ncbi.nlm.nih.gov/geo/). For single‐cell RNA sequencing (scRNA‐seq) analysis, the GSE149512 dataset [7] (including five control and three iNOA testicular samples) and the GSE112013 dataset [58] (three control testicular samples) were processed using the Seurat package (v.4.2.2). Cells were retained only if they met the following QC criteria: nCount_RNA >300, nFeature_RNA< 5,000, and mitochondrial gene percentage < 10%; all other cells were excluded as low‐quality cells. Data normalization and scaling were performed using the SCTransform function. To correct batch effects, the Harmony package was applied for data integration [59], and potential doublets were identified and removed using the DoubletFinder package [60]. Cell clusters were annotated using the established marker genes to identify the cell types [61]. The “GOBP_unsaturated_fatty_acid_ biosynthetic_process” gene ontology term was used to calculate the characteristic scores for each cell.

### 5.4 Immunohistochemistry and Immunofluorescence

#### 5.4.1 Immunohistochemistry

Frozen sections (after fixation) and paraffin sections (after deparaffinization, rehydration, and antigen retrieval) were treated with 3% H_2_O_2_ to quench endogenous peroxidase activity and blocked with 5% horse serum. Primary antibodies against FADS2, COL1A1, and p‐MLKL were incubated overnight at 4°C, followed by incubation with HRP‐conjugated secondary antibodies the next day. Signals were visualized with DAB, and nuclei were counterstained with hematoxylin. Staining intensity was independently evaluated by three pathologists.

#### 5.4.2 Immunofluorescence

Paraffin sections, after deparaffinization and antigen retrieval, were blocked and incubated with primary antibodies, including Claudin‐11, SYCP3, γH2A.X, DDX4, and TNP1, followed by incubation with fluorophore‐conjugated secondary antibodies (A11029, A11037, Invitrogen). Nuclei were counterstained with DAPI. Images were acquired using confocal microscopy. Co‐localization between FADS2 and STING was evaluated by calculating Pearson's correlation coefficients with ImageJ (v_1.8.0).

### 5.5 Western Blot

Testicular tissues or cells were lysed on ice in RIPA lysis buffer (P0038, Beyotime) containing protease and phosphatase inhibitors (P1045, Beyotime). Protein concentrations were determined using a BCA protein assay kit (23235, Thermo Fisher Scientific), and equal amounts of protein were mixed with loading buffer (P1005, Beyotime), separated by SDS‐PAGE, and transferred onto PVDF membranes (IPVH00010, Merck). After blocking with 5% non‐fat milk, the membranes were incubated with specific primary antibodies, followed by HRP‐conjugated secondary antibodies (SA00001‐2, SA00001‐1, Proteintech). Signals were visualized using an ECL chemiluminescent substrate kit (34580, Thermo Fisher Scientific) and captured with a chemiluminescence imaging system (Mini Chemi610, SINASGE, Beijing, China).

### 5.6 Primary Antibodies

Primary antibodies used in this study were anti‐GAPDH (10494‐1‐AP, Proteintech), anti‐FADS2 (68026‐1‐Ig, Proteintech), anti‐SCD (28678‐1‐AP, Proteintech), anti‐Claudin‐11 (sc‐271232, Santa Curz Biotechnology), anti‐Occludin (27260‐1‐AP, Proteintech), anti‐β‐catenin (8480, CellSignaling Technology), anti‐E‐cadherin (20874‐1‐AP, Proteintech), anti‐Vinculin (26520‐1‐AP, Proteintech), anti‐ Phospho‐STING (Ser365) (72971, CellSignaling Technology), anti‐STING (19851‐1‐AP, Proteintech), anti‐mouse‐ Phospho‐MLKL (Ser345) (ab196436, Abcam), anti‐human‐ Phospho‐MLKL (Ser358) (ab187091, Abcam), anti‐COL1A1 (ab255809, Abcam), anti‐COL1A2 (66761‐1‐IG, Proteintech), anti‐COL3A1 (HA720050, HUABIO), anti‐β‐actin (ET1607‐43, HUABIO), anti‐DDX4 (ab27591, Abcam), anti‐γH2A.X (ab26350, Abcam), anti‐SYCP3 (ab15093, Abcam), anti‐TNP1 (17178‐1‐Ig, Proteintech), anti‐SOX9 (ab185966, Abcam), anti‐Phospho‐STING (Ser366) (50907, CellSignaling Technology), anti‐Phospho‐TBK1 (Ser172) (5483, CellSignaling Technology), anti‐TBK1 (HA601045, HuaBio).

### 5.7 Quantitative Real‐Time PCR

Total RNA from testicular tissue and cultured cells was extracted using RNAex Pro Reagent (AG21102, Accurate Biology), and reverse transcribed into cDNA using a reverse transcription kit (11151ES60, Yeasen). qRT‐PCR was performed using a SYBR Green‐based system (11185ES08, Yeasen). Ribosomal protein lateral stalk subunit P0 (*Rplp0*) was used as the internal control. Relative expression levels were calculated using the 2^^−ΔΔCt^ method. Sequence of primer: *Fads2*‐Forward: 5'‐TCCTCTCGTACTTCGGCACT‐3', *Fads2*‐Reverse 5'‐GGTTCCACCAGTTGGCTGAG‐3', *Col1a1*‐Forward: 5'‐GTGTGTTCCCTACTCAGCCG‐3', *Col1a1*‐Reverse: 5'‐TGCTCTCTCCAAACCAGACG‐3'; *Col1a2*‐Forward: 5'‐GCTGAGGGCAACAGCAGGTTCACCTA‐3'; *Col1a2*‐Reverse: 5'‐GGAACGGCAGGCGAGATGGCTTATT‐3'; *Rplp0*‐Forward: 5'‐GGGCATCACCACGAAAATCTC‐3', *Rplp0*‐Reverse: 5'‐CTGCCGTTGTCAAACACCT‐3'; *FADS2*‐Forward: 5'‐GACCACGGCAAGAACTCAAAG‐3', *FADS2*‐Reverse: 5'‐GAGGGTAGGAATCCAGCCATT‐3'; *RPLP0*‐Forward: 5'‐GAAACTCTGCATTCTCGCTTC‐3', *RPLP0*‐Reverse: 5'‐GGTGTAATCCGTCTCCACAG‐3'.

### 5.8 Isolation and Identification of Primary Human Sertoli Cells

Primary human Sertoli cells were isolated from testicular tissue obtained from patients with obstructive azoospermia using a previously described two‐step enzymatic digestion method [62], with minor modifications. Enzyme solution I consisted of 2 mg/mL collagenase IV (WBC‐LS004188, Worthington) and 1 µg/µL DNase I (18047‐019, Gibco). Enzyme solution II consisted of 4 mg/mL collagenase IV, 2.5 mg/mL hyaluronidase (H3506, Sigma), 2 mg/mL trypsin (T4799, Sigma), and 1 µg/µL DNase I. Briefly, freshly obtained testicular tissues were washed three times with DMEM/F12 containing 2% penicillin‐streptomycin. The tissues were first digested with enzyme solution I in a 34°C water bath for 15 min to isolate seminiferous tubules. The seminiferous tubules were then digested with enzyme solution II in a 34°C water bath for 15 min and filtered through a 70 µm cell strainer to obtain a Sertoli cell‐enriched suspension. The cells were seeded in culture dishes and incubated overnight at 34°C in a humidified incubator containing 5% CO_2_. The medium was replaced the next day to remove non‐adherent cells. Primary human Sertoli cells were maintained in DMEM/F12 supplemented with 10% fetal bovine serum, 1% penicillin–streptomycin, and 1% ITS‐X supplement (41400045, Gibco). The above medium was diluted 5‐fold with DMEM/F12 prior to the experiments.

### 5.9 Cell Lines, Culture and Treatments

The mouse Sertoli cell line TM4 was cultured in DMEM/F12 medium supplemented with 5% horse serum (164215, Procell), 2.5% fetal bovine serum (FBS, A5256701, Gibco), and 1% penicillin‐streptomycin (15140122, Gibco). The GC‐1spg and GC‐2spd were cultured in DMEM (C11995500BT, Gibco) containing 10% FBS and 1% penicillin–streptomycin. All cells were maintained at 37°C in a humidified atmosphere with 5% CO_2_. For experimental treatments, TM4 cells were cultured in media containing 0.5% FBS to mimic nutrient‐restricted conditions.

To knock down *Fads2*, TM4 cells were transfected with siRNAs targeting mouse Fads2 using Lipofectamine RNAiMAX (13778150, Thermo Fisher Scientific), with non‐targeting siRNA as a control in Opti‐MEM (31985010, Thermo Fisher Scientific). Sixty hours after transfection, the culture medium was changed, after which cells were used for functional assays. Knockdown efficiency was confirmed by qRT‐PCR and Western blot. Sequence of siRNA: mouse‐si*Fads2*‐1: GCAAGAAGAAGCUGAAAUATT; mouse‐si*Fads2*‐2: GCAUUGAAUACCAGGAGAATT; mouse‐si*Fads2*‐3: GGGAGGAGAUUCAGAAGCATT.

For pharmacological treatment in TM4 cells: Fads2 inhibitor: 5, 10, 25, 35, or 50 µM SC26196 (S276482, Aladdin) for 24–60 h. STING agonist: 5–10 µM DMXAA (D129922, Aladdin), 2.5 µM diABZI (T5516, Targetmol), and 5 µM 2’3’‐cGAMP (T10065L, Targetmol) were used to activate STING signaling in TM4 cells. STING inhibitor: 1 µM H‐151 (T5674, Targetmol) was applied in combination with SC26196 or in Fads2‐knockdown conditions for rescue experiments. cGAS inhibitor: 2.5 µM RU.521 for 24 h. Polyunsaturated fatty acids (PUFAs): GLA (L111861, Aladdin), DHA (D100925, Aladdin), and ALA (L105575, Aladdin) were added at final concentrations typically ranging from 4 to 16 µM. IFN‐β: Recombinant mouse IFN‐β (HY‐P73131, MedChemExpress) was applied to GC‐1spg or GC‐2spd cells at defined concentrations (1, 5, 10, and 20 ng/mL) prior to heat shock (HS) treatment. Recombinant human IFN‐β (300‐02BC‐01 M, PeproTech) was applied to human testicular tissues at 20 ng/mL prior to HS treatment. For pharmacological treatments, hSCs were treated with 10 µM SC26196 or 2.5 µM diABZI for 48 h. For the rescue experiments, cells were first treated with 10 µM SC26196 or 2.5 µM diABZI for 24 h, after which 16 µM GLA and 4 µM DHA were added in the continued presence of the corresponding compound for an additional 24 h before subsequent analyses.

### 5.10 Cell Viability Assay (CCK‐8)

TM4 cells were seeded into 96‐well plates and subjected to pharmacological treatments. At indicated time points (24, 36, 48, and 60 h), Cell Counting Kit‐8 (CCK‐8) (FC101, Transgen Biotech) reagent was added to each well and incubated for 1 h. Absorbance at 450 nm was measured using a microplate reader. Cell viability was expressed as relative absorbance compared with the corresponding control group.

### 5.11 Measurement of Reactive Oxygen Species (ROS)

After the indicated treatments, TM4 cells were incubated with a ROS‐sensitive fluorescent probe (DCFH‐DA) (S0033S, Beyotime) at 37°C for 30 min according to the manufacturer's instructions, followed by washing with PBS. Samples were analyzed by flow cytometry. Appropriate forward and side scatter gating was used to exclude debris and doublets. Data were processed using FlowJo (FlowJo 10.8.1) to evaluate the percentage of ROS‐positive cells and assess the effects of siFads2, SC26196, DMXAA, H‐151, and PUFA treatments on oxidative stress.

### 5.12 TM4 Spheroid

After 24 h of transfection, TM4 cells were seeded into U‐bottom 96‐well plates (701111, NEST) to facilitate spontaneous aggregation and formation of 3D spheroids. Before use, U‐bottom 96‐well plates were treated with Anti‐Adherence Rinsing Solution (07010, StemCell Technologies) for 30 min and washed three times with PBS. At 72 h after transfection, TM4 spheroids were incubated with 10 µg/mL fluorescent microplastic particles (Fluo‐MPs) for 10 min. TM4 spheroids were fixed and processed for cryosectioning. Confocal microscopy was used to visualize the distribution of red Fluo‐MPs inside and outside the spheroids, thereby assessing barrier permeability. The images were captured using a confocal microscope (Nikon Al R).

### 5.13 Human Testicular Tissue Culture

Human testicular tissue was washed twice using PBS, sectioned into 2 mm pieces using sterile scissors, and placed on 1% agarose gel. Testicular tissue was maintained in spermatogonial stem cell culture medium (GCM‐H191, Procell) at 34°C, with the medium replaced every other day. Experimental reagents were added for 72 h, optionally followed by incubation with 10 µg/mL Fluo‐MPs for 10 min. At the end of treatment, tissue pieces were fixed, paraffin‐embedded, or cryosectioned for the next step of the experiment.

### 5.14 Sequencing of RNA and Protein

RNA‐seq of the TM4 sample was conducted for each sample using the NovaSeq‐PE150 platform at Novogene Co., Ltd. (Beijing, China). Protein sequencing of the TM4 sample was conducted on the Orbitrap Astral platform at Novogene Co., Ltd. (Beijing, China). Based on the UniProt protein database, the raw files were analyzed using the search software DIA‐NN. The differentially expressed genes (DEGs) or differentially abundant proteins (DAPs) were identified based on fold change, in conjunction with a *p*‐value. Gene ontology (GO) was conducted in Metascape (https://metascape.org/gp/).

### 5.15 ELISA

Supernatants from TM4 cell cultures or ex vivo human testicular tissue cultures were collected and centrifuged to remove cells and debris. IFN‐β levels were measured using mouse‐IFN‐β ELISA kit (E‐EL‐M0033, Elabscience) or human‐IFN‐β ELISA kit (E‐EL‐H0085, Elabscience) according to the manufacturer's instructions. Absorbance was read on a microplate reader, and concentrations were calculated from a standard curve.

### 5.16 Heat Shock and Necroptosis Assays

GC‐1spg and GC‐2spd cells were pretreated with various concentrations of recombinant IFN‐β or conditioned media from TM4 cells (after *Fads2* knockdown or FADS2 inhibition) for 18 h. Cells in 1.5 mL tube were then subjected to HS, typically at 43°C for 2 h in a water bath, followed by recovery at 37°C. Human testicular tissues were pretreated with IFN‐β or SC26196 for 48 h and then subjected to HS, typically at 43°C for 2 h in a water bath. Samples were harvested for experiments.

### 5.17 Multimodal SRS and SHG Image Acquisition

The stimulated Raman scattering and second harmonic generation microscopy system used in this study is based on the previously described system [32], as illustrated in Figure S8. A 1031 nm picosecond laser served as the Stokes beam, while a synchronously pumped optical parametric oscillator (OPO) generated a tunable picosecond output ranging from 700 to 990 nm, which served as the pump beam. The pump wavelength was tuned to excite Raman shifts at 2850 cm^−^^1^ (lipids) and 2930 cm^−^^1^ (proteins). The Stokes beam was intensity‐modulated at 80 MHz using an electro‐optic modulator and then combined collinearly with the pump beam via a dichroic mirror (650 nm cutoff; Thorlabs). The overlapped beams were directed into a laser‐scanning microscope (BX51WI, Olympus) and focused onto the sample through a water‐immersion objective (UPLSAPO 60XWIR, NA 1.2; Olympus). The average powers measured at the sample were approximately 58 mW for the pump beam and 74 mW for the Stokes beam. The transmitted pump beam was collected by a photodiode, and the SRS signal was demodulated using a lock‐in amplifier synchronized to the 20 MHz modulation frequency. Simultaneously, SHG signals from collagen fibers were collected in the epi‐direction through the same objective and detected by a photomultiplier tube (PMT). A 530/43 nm bandpass filter together with a 750 nm short‐pass filter was placed in front of the PMT to spectrally isolate the SHG signal. Each field of view (FOV) covered an area of 300 × 300 µm^2^ (400 × 400 pixels) with a pixel dwell time of 10 µs and was averaged three times. The lateral resolution of the system was approximately 300 nm. For visualization, SRS images acquired at 2850 and 2930 cm^−^^1^, together with SHG images, were pseudo‐colored using ImageJ.

### 5.18 Quantification of Collagen Thickness in SHG Images

Image processing was performed to quantify collagen fiber morphology from the SHG images. A binary mask of the collagen fibers was generated by applying a threshold to the original SHG grayscale images to isolate the fiber structures. Subsequent morphological analysis was performed using MATLAB (version R2023a). The skeletonization operation was performed on the binary mask to extract the central skeleton of each collagen fiber, representing its medial axis. A distance transform was then applied to the original mask, which assigned each pixel in the mask a value corresponding to the shortest distance to the background. The resulting skeleton was overlaid onto the distance map. For every pixel on the skeleton, the local fiber thickness was calculated as twice the corresponding distance transform value at that pixel location, representing the diameter of the fiber at that position. Finally, the maximum, average, and distribution of these thickness values were computed. For statistical comparison across seminiferous tubules, the median value of the collagen thickness within each tubule was selected as the representative metric.

### 5.19 Etiologic Classification of Azoospermia and Sperm Retrieval Outcomes

Clinical data from azoospermic patients who underwent testicular biopsy or micro‐TESE at our center between January 2021 and January 5, 2025 were retrospectively collected. Based on etiologic evaluation, patients were categorized into iNOA or non‐iNOA. Testicular histology was reviewed by experienced pathologists to determine the presence or absence of STBM thickening. Within the iNOA subgroup, sperm retrieval outcomes from micro‐TESE were compared between patients with and without basement membrane thickening.

### 5.20 Animal Treatment

Seven‐week‐old male C57BL/6J mice were purchased from Shanghai Model Organisms Center, Inc. (Shanghai, China). All animal experiments were performed in accordance with the guidelines of the Committee on Animal Research and Ethics and were approved by the Institutional Animal Care and Use Committee of Guangzhou Seyotin Biotechnology Co., Ltd. (Approval No. SYT2025038). After a one‐week acclimation, male mice were randomly assigned to three groups (*n* = 6 per group): Control, Busulfan, and Busulfan plus GLA and DHA. One day after a single intraperitoneal injection of 15 mg/kg busulfan, the mice showed no abnormalities and were then given GLA and DHA, which were prepared in 0.5% sodium carboxymethyl cellulose (CMC‐Na), by oral gavage at 150 mg/kg/day each. After 12 weeks of treatment, male mice were co‐housed with 10‐week‐old female mice at a 1:2 (male to female) ratio. Samples were collected after 5 days of co‐housing. The female mice were then maintained until parturition, and the pregnancy rate and litter size were recorded.

Seven‐week‐old male *Sting*‐knockout (KO) mice and their corresponding wild‐type (WT) control mice were purchased from Shanghai Model Organisms Center, Inc. (Shanghai, China). After a one‐week acclimation, male mice were randomly assigned to three groups (*n* = 6 per group): WT, WT plus Busulfan, and *Sting* KO plus Busulfan. Eight weeks after a single intraperitoneal injection of busulfan at 15 mg/kg, testicular samples were collected for analysis.

Seven‐week‐old male C57BL/6J mice were purchased from GemPharmatech Co., Ltd. (China). After a one‐week acclimation, male mice were randomly assigned to four groups (*n* = 6 per group): Control, SC26196, SC26196 plus ALA, and SC26196 plus GLA and DHA. The SC26196, ALA, GLA, and DHA were prepared in 0.5% CMC‐Na. Mice in the SC26196, SC26196 plus ALA, and SC26196 plus GLA and DHA groups were given 100 mg/kg/day SC26196 by oral gavage. Mice in the SC26196 plus ALA group were additionally administered ALA by oral gavage at 150 mg/kg/day, whereas mice in the SC26196 plus GLA and DHA group were additionally administered GLA and DHA by oral gavage at 150 mg/kg/day each. After 8 weeks of treatment, testicular tissues were collected for analysis.

### 5.21 Sperm Parameters Measurement

Sperm parameters were evaluated as previously described [63]. Epididymides were collected from each mouse and placed into HTF medium. After carefully trimming away the surrounding adipose tissue, the epididymides were chopped into small fragments with micro scissors. The tissue pieces were then incubated at 37°C for 5 min, followed by further disruption in pre‐warmed G‐IVF PLUS medium (10136, Vitrolife, Sweden) at 37°C to release sperm into the medium. The resulting sperm suspension was incubated for an additional 15 min at 37°C to allow uniform dispersion, after which sperm concentration and motility were evaluated using a computer‐assisted sperm analysis (CASA) system.

### 5.22 Statistical Analysis

Data are presented as mean ± standard deviation (SD) or mean ± standard error of the mean (SEM), as indicated in the Figure legends. For statistical comparisons, one‐way ANOVA and unpaired Student's *t*‐tests were employed. Categorical variables (e.g., proportion of tubules with basement membrane thickening, sperm retrieval success rates) were compared using χ^2^ tests. Correlation analyses were performed using Pearson or Spearman correlation coefficients, as specified in the Figure legends. Binary logistic regression analysis was performed using SPSS version 26.0 to evaluate the association between STBM thickening and successful sperm retrieval. The crude model was unadjusted. Model 1 was adjusted for age and BMI. Model 2 was adjusted for age, BMI, E_2_, FSH, LH, PRL, and T. Odds ratios (ORs) and 95% confidence intervals (CIs) were reported. *p*‐value < 0.05 was considered statistically significant.

## Author Contributions

Conceptualization: **Geng An**, **Zhicong Chen**, and **Hyeon Jeong Lee**; methodology: **Jiajing Lin, Hongfei Ke**, and **Zhengyan Wu**; investigation: **Jiajing Lin**, **Hongfei Ke**, and **Xiaomin Li**; writing – original draft: **Jiajing Lin**, **Xiaomin Li**, and **Man Wu**; writing – review and editing: **Jiajing Lin**, **Xiaomin Li**, **Man Wu**, **Hyeon Jeong Lee**, **Zhicong Chen**, and **Geng An**; formal analysis: **Jiajing Lin** and **Hongfei Ke**; resources: **Jiajing Lin**, **Hongfei Ke**, **Zhengyan Wu**, **Tianwen Peng**, **Yu Lan**, **Xin Fu**, and **Yingying Zhang**; validation: **Xiaomin Li**, **Man Wu**, **Zhiying Huang**, **Feixue Han**, **Shuyan Chen**, and **Dejian Pang**; funding acquisition: **Geng An** and **Zhicong Chen**; supervision: **Geng An**.

## Conflicts of Interest

The authors declare no conflicts of interest.

## Supporting information




**Supporting File**: advs78583‐sup‐0001‐SuppMat.docx.

## Data Availability

The transcriptomics data generated in this study have been deposited in the NCBI BioProject database (PRJNA1480192). The proteomics data generated in this study have been deposited in the ProteomeXchange Consortium via the iProX partner repository (PXD079976). The lipidomics data reported in this paper have been deposited in the OMIX (OMIX018557), China National Center for Bioinformation / Beijing Institute of Genomics, Chinese Academy of Sciences (https://ngdc.cncb.ac.cn/omix). Other data supporting the findings of this study are available from the corresponding author upon reasonable request.
