# Supplementary material for: FADS2‐STING Signaling in Sertoli Cells Orchestrates Collagen Deposition and Germ Cell Necroptosis in Idiopathic Non‐Obstructive Azoospermia
Source: Adv Sci (Weinh). 2026 Nov 9:e78583. Online ahead of print. doi: 10.1002/advs.78583 (PMC13748773; doi:10.1002/advs.78583)
Supplement: Supplementary file 1 [file ADVS-9999-e78583-s001.docx]

Supporting Information

**FADS2-STING signaling in Sertoli cells orchestrates collagen deposition and germ cell necroptosis in idiopathic non-obstructive azoospermia**

Jiajing Lin^2^, Hongfei Ke^1,2^, Xiaomin Li^2^, Man Wu^2^, Zhengyan Wu^3^, Zhiying Huang^2^, Feixue Han^2^, Dejian Pang^2^, Shuyan Chen^4^, Tianwen Peng^2^, Yu Lan^2^, Xin Fu^2^, Yingying Zhang^2^, Hyeon Jeong Lee^3^*, Zhicong Chen^2^*, Geng An^1,2^*

^1^National Health Commission (NHC) Key Laboratory of Male Reproduction and Genetics, Guangdong Provincial Reproductive Science Institute (Guangdong Provincial Fertility Hospital), Guangzhou 510600, China

^2^Department of Obstetrics and Gynecology, Center for Reproductive Medicine, Guangdong Provincial Key Laboratory of Major Obstetric Diseases, Guangdong Provincial clinical Research Center for Obstetrics and Gynecology, Guangdong Hong Kong-Macao Greater Bay Area Higher Education Joint Laboratory of Maternal-Fetal Medicine, The Third Affiliated Hospital, Guangzhou Medical University, Guangzhou 510150, China

^3^College of Biomedical Engineering & Instrument Science, Key Laboratory for Biomedical Engineering of Ministry of Education, Zhejiang University, Hangzhou 310058, China

^4^The First People's Hospital of Zhaoqing, Zhaoqing 526000, China

*Correspondence:Hyeon Jeong Lee ([hjlee@zju.edu.cn](mailto:hjlee@zju.edu.cn)); Zhicong Chen (zcchen@gzhmu.edu.cn); Geng An (angeng0505@outlook.com)

**Figure S1. Lipidomic analyses of control and iNOA testes.** A) Partial least squares discrimination analysis (PLS-DA) of lipidomic from human testicular tissue (Control, *n* = 11; iNOA, *n* = 17). Showing the relationship between the expression levels of lipid compounds and sample categories. B) Volcano plots for lipidomic in human testicular tissue (control group, *n* = 11; iNOA group, *n* = 17). Significance thresholds: Log_2_FC > 1, *P* < 0.05, VIP > 1. C) Total percentage of lipids containing each fatty acyl chain in control (*n* = 11) and iNOA group (*n* = 17). Data are expressed as mean ± SEM. *P* values were determined by Student's t-test. **P* < 0.05, ****P* < 0.001, n.s, not significant.

**Figure S2. Single-cell transcriptomic analyses of control and iNOA testes.** A-B) UMAP plot showing integration of scRNA-seq data with GSE149512 and GSE112013, containing 8 Control samples and 3 iNOA samples (A). UMAP plots showing the cell colors by the identified cell clusters (B). C) Dot plot of expression patterns of selected markers. Dot Color represents the average expression level. Dot size represents the percentage of expressing cell. Leydig/PTM: Leydig cell and Peritubular myoid cell (PTM); M.Leydig: Mature Leydig cell; Sertoli: Sertoli cells; diff.ing. SPG: Differentiating/Intermediate Spermatogonia; Div. SPC: Dividing Spermatocytes; Pachy SPC: Pachytene Spermatocytes; rSPT: Round Spermatids; eSPT: Elongating/Elongated Spermatids; Late SPT: Late Spermatids / Spermatozoa-like cells; CD8 T: CD8⁺ T cells; Mac: Macrophage; Endo: Endothelial cells; Mast: Mast cells.

**Figure S3. Identification of primary human Sertoli cells (hSCs)**. A) Bright-field images and immunofluorescence characterization (SOX9) of passage 3 hSCs. B) Expression levels of FADS2 and STING in TM4 cells and hSCs.

**Figure S4. FADS2 inhibition affects TM4 cellular functions.** A-B) Validation of *Fads2* knockdown in TM4 cells by qRT-PCR (A) and Western blot (B). C-D) Flow cytometry histograms of ROS-positive TM4 cells after *Fads2* knockdown (C) or FADS2 inhibition (D). E) Cell viability at 36, 48 and 60 h in TM4 cells treated with 10μM SC26196 in the presence of 4 μM GLA or 4 μM ALA. F) Cell viability at 36, 48 and 60 h in TM4 treated with SC26196 in the presence of 4 μM GLA or 4 μM GLA and 4 μM DHA. G-H) Representative immunofluorescence images (G) of Fluo-MPs (red) penetration in the TM4 spheroid after FADS2 inhibition and quantification of relative area of Fluo-MPs to DAPI (H). Scale bars indicate 100 μm. I-J) Relative quantification of the Western blot bands shown in Figure 2O. Data are expressed as mean ± SD of three independent experiments. *P* values were determined by Student's t-test in (A, B, H, I and J) and one-way ANOVA in ( E and F) . ***P* < 0.01, ****P* < 0.001, n.s, not significant.

**Figure S5. Supporting evidence for STING activation upon Fads2 loss in TM4 cells.** A) Volcano plots for transcriptomics (left) and proteomics (right) in *Fads2*-knockdown TM4 cells. Venn diagram showing the overlap between differentially expressed genes (DEGs) and differentially abundant proteins (DAPs). Significance thresholds: transcriptomics: FC > 1.2 or < 0.833 and *P* < 0.05. B) Western blot analysis and quantification of TBK1, p-TBK1 (Ser172), STING and p-STING (Ser365) in *Fads2*-knockdown TM4 cells. C) Relative quantification of the Western blot bands shown in Figure 3B. D) Western blot analysis and quantification of TBK1, p-TBK1 (Ser172), STING and p-STING (Ser365) in TM4 cells following treatment of SC26196, ALA or GLA. E) Relative quantification of the TM4 cells Western blot bands shown in Figure 3E. F-G) Representative immunofluorescence images and Pearson's correlation coefficient values for co-localization of FADS2 and STING in TM4 cells treated with SC26196 or GLA and DHA, staining FADS2 (green) and STING (red). H) Western blot analysis and quantification of TBK1, p-TBK1 (Ser172), STING and p-STING (Ser365) in TM4 cells treated with SC26196, RU.521 or GLA and DHA. I) Relative quantification of the TM4 cells Western blot bands shown in Figure 3G. J) Representative immunofluorescence images and Pearson's correlation coefficient values for co-localization of FADS2 and STING in TM4 cells treated with SC26196 in the presence of H-151, staining FADS2 (green) and STING (red). Scale bars indicate 50 μm. Data are expressed as mean ± SD of three experiments. *P* values were determined by Student's t-test in (B and C) and one-way ANOVA in (D, E, G, H, I and J). **P* < 0.05, ***P* < 0.01, ****P* < 0.001, n.s, not significant.

**Figure S6. GLA and DHA supplementation attenuates STING agonist-induced STING pathway activation in Sertoli cells**. A) Cell viability at 36, 48 and 60 h in TM4 cells treated with 5 or 10 μM DMXAA. B) Flow cytometry and quantification of ROS levels in TM4 cells treated with 5 μM DMXAA for 60 h. C) Representative immunofluorescence images and Pearson's correlation coefficient values for co-localization of FADS2 and STING in TM4 cells treated with DMXAA in the presence of GLA and DHA, staining FADS2 (green) and STING (red). Scale bars indicate 50 μm. D) Relative quantification of the Western blot bands shown in Figure 3O. Data are expressed as mean ± SD. *P* values were determined by Student's t-test in (B) and one-way ANOVA in (A, C and D). **P* < 0.05, ***P* < 0.01, ****P* < 0.001, n.s, not significant.

**Figure S7. GLA and DHA supplementation attenuates SC26196-induced testicular injury and STING pathway activation in vivo**. A) Schematic diagram of the animal experiments. B-C) Sperm density (million/mL) (B) and sperm motility (%) (C) across groups (*n* = 6 per group). D) Representative hematoxylin and eosin (H&E) staining images across groups. E) Western blot analysis and quantification of TBK1, p-TBK1 (Ser172), STING and p-STING (Ser365) in mouse testicular tissues from each group. Scale bars indicate 100 μm. Data are expressed as mean ± SD. *P* values were determined by one-way ANOVA. **P* < 0.05, ***P* < 0.01, ****P* < 0.001, n.s, not significant.

**Figure S8. Schematic representation of SRS/SHG microscopy experimental setup.**

EOM: electro-optic modulator; DM: dichroic mirror; SU: scanning unit; F: filter; PD: photodiode; PMT: photomultiplier tube; TIA: Transimpedance Amplifier.

**Figure S9. FADS2 depletion and STING activation increase collagen synthesis.** A) Volcano plots for transcriptomics in DMXAA stimulated TM4 cells. B-D) Collagen related gene or protein expression in TM4 cells, DMXAA group vs DMSO group (B), *Fads2*-knockdown group vs control group (C-D). E) Western blot analysis of COL1A1, COL1A2, TBK1, p-TBK1 (Ser172), STING and p-STING (Ser365/366) in TM4 treated with STING agonist (DMXAA, diABZI and 2’3’-cGAMP) or GLA and DHA, and in hSCs treated with SC26196, diABZI or GLA and DHA. F) Relative mRNA expression of *Col1a1* and *Col1a2* in FADS2-inhibited TM4 cells with or without H-151 treatment. G) Relative quantification of the Western blot bands shown in Figure 5I. H-I) Relative mRNA expression of *Col1a1* and *Col1a2* in TM4 treated with SC26196 (H) or DMXAA (I), with supplementation of GLA and DHA. J) Western blot analysis of TBK1, p-TBK1 (Ser172), STING, p-STING (Ser365) COL1A1 and COL1A2 in TM4 treated STING agonist ( diABZI and 2’3’-cGAMP) or GLA and DHA. K) Relative mRNA expression of *COL1A1* and *COL1A2* of human testicular tissue after treatment of SC26196 combined with H-151 or GLA and DHA. L) Representative Immunohistochemistry images of human testicular tissues in ex vivo treated with SC26196, GLA and DHA. Scale bars indicate 100 μm. Data are expressed as mean ± SD of three independent experiments. *P* values in (F, G, H, I and K) were determined by one-way ANOVA. * *P* < 0.05, ** *P* < 0.01, ****P* < 0.001.

**Figure S10. STING deficiency attenuates busulfan-induced testicular injury.** A) Schematic diagram of the animal experiments. B-C) Sperm density (million/mL) (B) and sperm motility (%) (C) among group (*n* = 6 per group). D) Representative hematoxylin and eosin (H&E) and Masson staining images across groups. E) Quantification of seminiferous tubules exhibiting partial or full spermatogenesis based on H&E staining images. Scale bars indicate 100 μm. F) Western blot analysis of TBK1, p-TBK1 (Ser172), STING and p-STING (Ser365) in mouse testicular tissues from wild type (WT) and *Sting*-knockout (KO) mice treated with busulfan. G) Western blot analysis of p-MLKL, MLKL and FADS2 in mouse testicular tissues across groups (Control, Busulfan, Busulfan plus GLA and DHA). Data are expressed as mean ± SEM. *P* values in (B and C) were determined by one-way ANOVA. * *P* < 0.05, ****P* < 0.001, n.s, not significant.

**Table S1. Baseline characteristics of iNOA stratified by STBM thickening status**

| **Variable** | **Non-thickened STBM (*n* = 163)** | **Thickened STBM**  **(*n* = 445)** | ***P* value** |
| --- | --- | --- | --- |
| Age (years) | 31.00 [28.00, 34.00] | 31.00 [29.00, 34.00] | 0.388 |
| BMI (kg/m²) | 23.25 [21.30, 26.03] | 24.20 [22.00, 26.10] | 0.239 |
| E_2_ (pmol/L) | 68.00 [47.25, 100.00] | 76.00 [53.00, 103.00] | 0.047 |
| FSH (IU/L) | 19.02 [11.93, 26.98] | 18.63 [12.94, 24.33] | 0.436 |
| LH (IU/L) | 7.26 [4.82, 10.34] | 6.63 [4.68, 9.26] | 0.133 |
| PRL (ng/mL) | 10.92 [8.14, 15.08] | 10.78 [7.92, 15.03] | 0.616 |
| T (nmol/L) | 11.58 [8.10, 17.91] | 11.74 [7.99, 16.95] | 0.788 |
| Sperm retrieval, n (%) | 61 (37.42) | 95 (21.35) | <0.001 |

* Data are presented as median (interquartile range) for continuous variables and n (%) for categorical variables. Analyses were performed using available non-missing data for each variable. Continuous variables were compared using the Mann-Whitney U test, and categorical variables were compared using the χ² test. BMI, body mass index; CI, confidence interval; E₂, estradiol; FSH, follicle-stimulating hormone; LH, luteinizing hormone; OR, odds ratio; PRL, prolactin; STBM, seminiferous tubule basement membrane; T, testosterone.

**Table S2. Logistic regression analysis of the association between STBM thickening and successful sperm retrieval**

| **Model** | **N** | **OR** | **95% CI** | ***P* value** |
| --- | --- | --- | --- | --- |
| Crude Model | 608 | 0.454 | 0.307-0.670 | <0.001 |
| Model 1 ^a^ | 608 | 0.465 | 0.312-0.693 | <0.001 |
| Model 2 ^b^ | 539 | 0.482 | 0.313-0.743 | 0.001 |

*Logistic regression models were fitted using complete-case data according to the variables included in each model. The odds ratios refer to the odds of successful sperm retrieval in patients with thickened STBM compared with those without STBM thickening.

^a^ Adjusted for age and BMI.

^b^ Adjusted for age, BMI, E_2_, FSH, LH, PRL, and T.

**Table S3. Cell numbers for each sample in scRNA-seq datasets.**

| Dataset | Sample/Donor | Input Cells | Retained Cells |
| --- | --- | --- | --- |
| GSE112013 | Donor 1 | 2248 | 2174 |
|  | Donor 2 | 1994 | 1939 |
|  | Donor 3 | 2248 | 2180 |
| GSE149512 | GSM4504182 | 6900 | 6589 |
|  | GSM4504185 | 10440 | 9578 |
|  | GSM4504191 | 3225 | 3169 |
|  | GSM4504192 | 3477 | 3412 |
|  | GSM4504193 | 3555 | 3492 |
|  | GSM4504195 | 7607 | 7391 |
|  | GSM4504196 | 6733 | 6535 |
|  | GSM4504197 | 6878 | 6570 |
